# The Key Glycolytic Enzyme Phosphofructokinase Is Involved in Resistance to Antiplasmodial Glycosides

**DOI:** 10.1128/mBio.02842-20

**Published:** 2020-12-08

**Authors:** Gillian M. Fisher, Simon A. Cobbold, Andrew Jezewski, Emma F. Carpenter, Megan Arnold, Annie N. Cowell, Erick T. Tjhin, Kevin J. Saliba, Tina S. Skinner-Adams, Marcus C. S. Lee, Audrey Odom John, Elizabeth A. Winzeler, Malcolm J. McConville, Sally-Ann Poulsen, Katherine T. Andrews

**Affiliations:** a Griffith Institute for Drug Discovery, Griffith University, Queensland, Australia; b University of Melbourne, Victoria, Australia; c University of California, San Diego, San Diego, California, USA; d Washington School of Medicine, St. Louis, Missouri, USA; e Wellcome Sanger Institute, Cambridge, United Kingdom; f The Australian National University, Canberra, Australia; g Children’s Hospital of Philadelphia, Philadelphia, Pennsylvania, USA; National Institute of Allergy and Infectious Diseases

**Keywords:** *Plasmodium falciparum*, drug resistance mechanisms, drug targets, glycolysis, metabolic regulation

## Abstract

*Plasmodium* parasites rely heavily on glycolysis for ATP production and for precursors for essential anabolic pathways, such as the methylerythritol phosphate (MEP) pathway. Here, we show that mutations in the Plasmodium falciparum glycolytic enzyme, phosphofructokinase (*Pf*PFK9), are associated with *in vitro* resistance to a primary sulfonamide glycoside (PS-3). Flux through the upper glycolysis pathway was significantly reduced in PS-3-resistant parasites, which was associated with reduced ATP levels but increased flux into the pentose phosphate pathway. PS-3 may directly or indirectly target enzymes in these pathways, as PS-3-treated parasites had elevated levels of glycolytic and tricarboxylic acid (TCA) cycle intermediates. PS-3 resistance also led to reduced MEP pathway intermediates, and PS-3-resistant parasites were hypersensitive to the MEP pathway inhibitor, fosmidomycin. Overall, this study suggests that PS-3 disrupts core pathways in central carbon metabolism, which is compensated for by mutations in *Pf*PFK9, highlighting a novel metabolic drug resistance mechanism in P. falciparum.

## Abstract (precis)

Malaria, caused by *Plasmodium* parasites, continues to be a devastating global health issue, causing 405,000 deaths and 228 million cases in 2018. Understanding key metabolic processes in malaria parasites is critical to the development of new drugs to combat this major infectious disease. The *Plasmodium* glycolytic pathway is essential to the malaria parasite, providing energy for growth and replication and supplying important biomolecules for other essential *Plasmodium* anabolic pathways. Despite this overreliance on glycolysis, no current drugs target glycolysis, and there is a paucity of information on critical glycolysis targets. Our work addresses this unmet need, providing new mechanistic insights into this key pathway.

## INTRODUCTION

The parasite Plasmodium falciparum is the major cause of malaria-associated mortality, with an estimated 405,000 deaths reported in 2018 (1). Unfortunately, there is no broadly effective malaria vaccine, and increasing resistance to current antimalarials is driving the search for new targets for antimalarial drug development (1, 2). The malaria parasite has a complex life cycle, with the asexual intraerythrocytic stage being the cause of the clinical manifestations associated with this disease. During this stage, the malaria parasite relies heavily on glycolysis for ATP energy production, with glucose consumption increasing 100-fold in P. falciparum-infected erythrocytes (3). The first rate-limiting and regulatory step in the glycolytic pathway is the conversion of fructose-6-phosphate to fructose-1,6-biphosphate (FBP) by the enzyme phosphofructokinase (PFK). The P. falciparum genome contains two ATP-dependent PFK genes, *Pfpfk9* and *Pfpfk11*. *Pfpfk9* (PF3D7_0915400; chromosome 9 [4]) encodes a 160-kDa protein containing fused β and α domains that is structurally similar to plant PFKs and has low amino acid similarity to human PFKs (∼15%) (5). *Pfpfk9* has been shown to encode a catalytically active PFK enzyme (5) and appears to be essential (6). In contrast, *Pfpfk11* (PF3D7_1128300; chromosome 11 [4]) appears to be dispensable in asexual-stage P. falciparum (6).

In recent work, *Pf*PFK9 mutations have been associated with the reversal of fosmidomycin resistance in P. falciparum (7). Fosmidomycin, an antibiotic and antimalarial drug candidate, acts as a competitive inhibitor of a key enzyme in methylerythritol phosphate (MEP) pathway isoprenoid biosynthesis in the apicoplast of the malaria parasite (8). Fosmidomycin resistance has been linked to the functional loss of haloacid dehalogenase 1 (*Pf*HAD1), a sugar phosphatase (9). In fosmidomycin-resistant parasites, loss of *Pf*HAD1 function results in dysregulation of glycolysis and increased flux of triose-phosphates into the MEP pathway, with a concomitant reduction in the effectiveness of fosmidomycin (9). A second HAD family member, *Pf*HAD2, has also been implicated in fosmidomycin resistance (7). Curiously, fosmidomycin sensitivity is restored in *Pf*HAD2 mutants that have been subjected to prolonged culture in the absence of drug pressure. These *Pf*HAD2 mutants gain mutations in *Pf*PFK9 that result in the restoration of fosmidomycin sensitivity (7).

**FIG 1:**
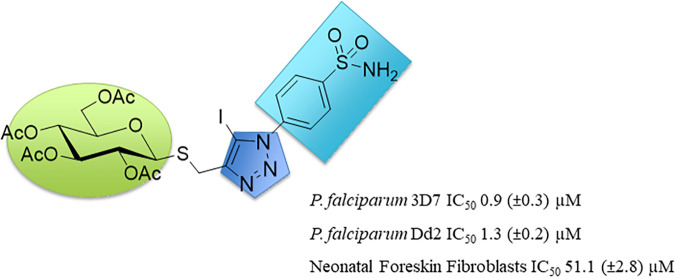
PS-3 structure and activity. The thioglucosyl moiety (S or sulfur at the anomeric position) of PS-3 is shown in green, and the primary sulfonamide in light blue. PS-3 contains an acetylated glucose group joined to a benzene sulfonamide via a triazole linker (dark blue). P. falciparum 3D7/Dd2 and neonatal foreskin fibroblast (NFF) IC_50_ values are as previously reported (10).

In this study, we provide the first report that *Pf*PFK9 mutations are associated with resistance to an antiplasmodial compound. Previously, we screened a panel of primary sulfonamide glycosides for *in vitro* growth inhibitory activity against asexual intraerythrocytic stages of drug-sensitive (3D7) and multidrug-resistant (Dd2) P. falciparum parasites. The most potent sulfonamide glycoside (PS-3) exhibited a 50% inhibitory concentration (IC_50_) value of ∼1 μM and >40-fold selectivity for P. falciparum versus that for mammalian cells (Fig. 1) (10). In this study, P. falciparum parasites were selected for *in vitro* resistance to PS-3 (10), followed by whole-genome sequence analysis of clones to identify putative PS-3 targets and/or resistance mechanisms. A point mutation in the *Pf*PFK9 gene was shown to be linked to PS-3 resistance and subsequently confirmed via reverse genetics. While PS-3 did not significantly inhibit recombinant *Plasmodium* PFK activity, PS-3-resistant P. falciparum parasites with *Pf*PFK9 gene mutations redirected glucose flux into the pentose phosphate pathway, at the expense of upper glycolysis, while still maintaining lower glycolysis and continued ATP production. While the precise target of PS-3 remains undefined, these data suggest that the redirection of carbon fluxes into the pentose phosphate pathway, mediated by mutations to *Pf*PFK9, confers resistance to this novel antiplasmodial compound.

## RESULTS

### Generation of PS-3-resistant P. falciparum clones.

**FIG 2:**
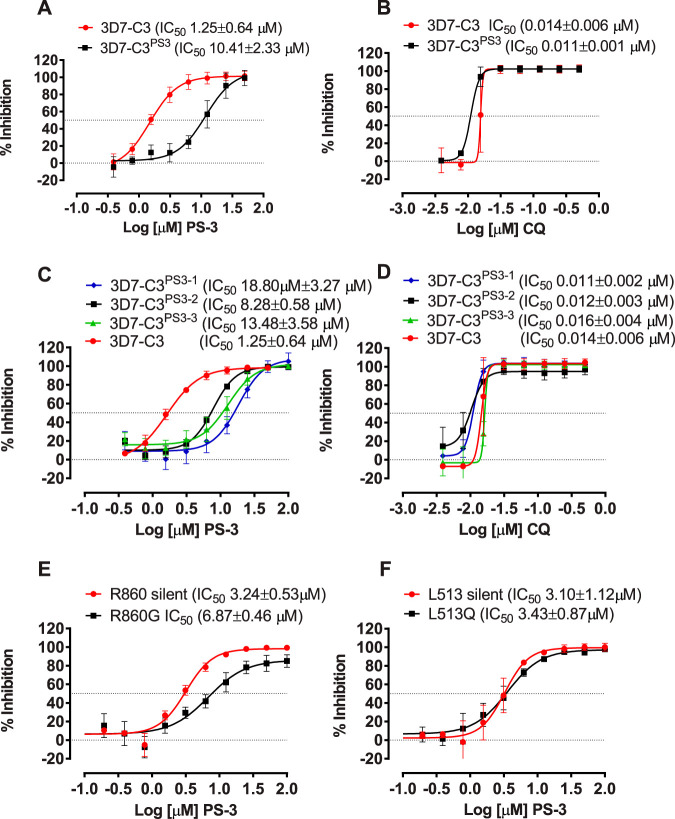
*In vitro* profiles of P. falciparum PS-3 resistant parasites, subclones, and *Pf*PFK mutants. The sensitivity of P. falciparum 3D7-C3^PS3^ versus that of wild type clone 3D7-C3 was assessed against compound PS-3 (A) and chloroquine (B). The sensitivity of P. falciparum 3D7-C3^PS3^ subclones (3D7-C3^PS3-1^, 3D7-C3^PS3-2^, and 3D7-C3^PS3-3^) and 3D7-C3 was assessed against PS-3 (C) and chloroquine (D). The sensitivity of P. falciparum PFK9 mutant lines R860G/R860 silent (E) and L513Q/L513 silent (F) was assessed against PS-3. In each case, the mean percent inhibition (± standard deviation [SD]) compared to that for DMSO controls was determined using 72-h [^3^H]hypoxanthine growth inhibition assays for at least three independent assays, each carried out in triplicate wells. Mean (±SD) 50% inhibitory concentrations (IC_50s_) were determined using nonlinear regression analysis in GraphPad prism.

A P. falciparum 3D7 clone was generated by limiting dilution (termed 3D7-C3) and used to generate PS-3-resistant parasites using a stepwise selection method, beginning at ∼1× IC_50_ (1 μM). After ∼80 asexual intraerythrocytic cycles, parasites were selected with the ability to grow in ∼10× the P. falciparum 3D7 wild-type PS-3 IC_50_ (∼10 μM; termed 3D7-C3^PS3^) (Fig. 2A). In contrast, there was no significant difference in IC_50_ values for the control drug chloroquine (*P* > 0.05) (Fig. 2B). The 3D7-C3^PS3^ line remained resistant to PS-3 following cryopreservation, thawing, and reculture (see Fig. S1A in the supplemental material), and this phenotype was stable after removal of PS-3 selection pressure for >10 weeks (Fig. S1B). Exposure of 3D7-C3^PS3^ parasites to higher concentrations of PS-3 (∼20× IC_50_ [20 μM]) for >8 weeks did not result in a significant alteration in the IC_50_ (*P* > 0.05) compared to that for 3D7-C3^PS3^ selected with 10× PS-3 (see Fig. S2). A comparison of *in vitro* IC_50_ values for 3D7-C3^PS3^ versus those for 3D7-C3 wild-type parasites showed generally no significant difference for the clinically used antimalarial drugs chloroquine, pyrimethamine, cycloguanil, artesunate, atovaquone, and quinine (*P* > 0.05) (Table 1). Likewise, there was no significant difference in IC_50_ values for the clinical candidates KAE609 (11) (*Pf*ATP4 inhibitor) and DSM161 (12) (an analogue of DSM265, a dihydroorotate reductase [DHOD] inhibitor [13]). This is reflected in the calculated resistance indices (Ri), which range from 0.8 to 1.6. In contrast, a Ri of 8.3 was obtained when comparing P. falciparum 3D7-C3 and 3D7-C3^PS3^ PS-3 IC_50_ values (Table 1).

**TABLE 1:** Activity of antiplasmodial compounds against P. falciparum 3D7-C3^PS3^ and 3D7-C3 parasites

Compound	IC_50_ (μM)^*a*^		Ri^*b*^	*P* value^*c*^
	3D7-C3 wild type	3D7-C3^PS3^		
PS-3	1.250 (±0.640)	10.410 (±1.380)	8.3	0.0006
Chloroquine	0.014 (±0.003)	0.011 (±0.004)	0.8	0.9142
Pyrimethamine	0.033 (±0.010)	0.054 (±0.028)	1.6	0.2826
Artesunate	0.004 (±0.002)	0.004 (±0.001)	1.0	0.6779
DSM161	0.345 (±0.065)	0.359 (±0.027)	1.0	0.7535
KAE609	0.0015 (±0.0001)	0.0014 (±0.0003)	0.9	0.5734
Atovaquone	0.0002 (±0.0001)	0.0002 (±0.0001)	1.0	0.5185
Cycloguanil	0.0103 (±0.0004)	0.0141 (±0.0002)	1.4	0.0001
Quinine	0.0224 (±0.0013)	0.0133 (±0.0003)	0.6	0.0101

a Mean IC_50_ (± SD) for three independent experiments, each in triplicate wells.

b Ri, resistance index: IC_50_ resistant line (3D7-C3^PS3^)/IC_50_ wild-type line (3D7-C3). The higher the Ri the, higher the level of resistance.

c Statistical difference between IC_50_s was determined using an unpaired *t* test with GraphPad Prism data analysis software.

10.1128/mBio.02842-20.1 FIG S1 P. falciparum 3D7-C3^PS3^ displays a stable phenotype. (A) The sensitivity of P. falciparum 3D7-C3^PS3^ to PS-3 prior to (black line) and after (red line) cryopreservation was assessed using 72-h [^3^H]hypoxanthine uptake growth inhibition assays. Mean percentage inhibition (±SD) is shown for three independent assays, each carried out in triplicate wells. (B) The sensitivity of P. falciparum 3D7-C3^PS3^ to PS-3 following withdrawal from PS-3 pressure for 4 weeks (blue line) and 10 weeks (red line) was assessed using 72-h [^3^H]hypoxanthine uptake growth inhibition assays and compared to the sensitivity of P. falciparum 3D7-C3^PS3^ exposed continually to PS-3 (10 μM; black line). Download FIG S1, PDF file, 0.3 MB. Copyright © 2020 Fisher et al. 2020 Fisher et al. This content is distributed under the terms of the Creative Commons Attribution 4.0 International license.

10.1128/mBio.02842-20.2 FIG S2 Exposure of 3D7-C3^PS3^ to ∼20× PS-3 IC_50_ does not significantly alter PS-3 activity. The sensitivity of P. falciparum 3D7-C3^PS3^ to PS-3 at ∼10× IC_50_ (10 μM; red line) and ∼20× IC_50_ (20 μM; black line) was assessed using 72-h [^3^H]hypoxanthine uptake growth inhibition assays. Mean percentage inhibition (±SD) is shown from three independent assays, each carried out in triplicate wells. Increasing PS-3 exposure from 10 μM to 20 μM did not result in any significant difference in 3D7-C3^PS3^ PS-3 IC_50_ (*P* > 0.05). Download FIG S2, PDF file, 0.2 MB. Copyright © 2020 Fisher et al. 2020 Fisher et al. This content is distributed under the terms of the Creative Commons Attribution 4.0 International license.

### Structure-activity relationship analysis of the P. falciparum PS-3 resistance phenotype.

To define which PS-3 structural group(s) contributes to the 3D7-C3^PS3^ resistance phenotype, PS-3 analogues were tested in *in vitro* growth inhibition assays against 3D7-C3 and 3D7-C3^PS3^ parasites. Compounds included PS-3′, which lacks the primary sulfonamide (PS) moiety, and four compounds that retain the PS moiety (PS-1, PS-3, PS-7, and PS-10) (Table 2). All compounds contain a per-*O*-acetylated glucose type sugar with an acetyl R group and a triazole substituent (Y = I or H) but vary with respect to their glycosidic linkage (X = S, O, or SO_2_) (Table 2). Compared to PS-3, the absence of the PS moiety in PS-3′ resulted in a significant reduction in activity for both 3D7-C3^PS3^ and 3D7-C3 wild-type parasites (∼4-fold higher IC_50_s; *P* < 0.05) (Table 2); however, the PS-3′ Ri remained similar (Ri, ∼6) (Table 2) to that for the PS-3 selection compound, indicating that the PS-3 resistance phenotype is independent of the PS group. Likewise, PS-7 and PS-10 showed a reduction in overall activity against both 3D7-C3^PS3^ and wild-type 3D7-C3 but retained similar Ri values to that for PS-3 (Ri ∼6 to 7) (Table 2), indicating that the variable glycosidic linkage (X = S, O, or SO_2_) (Table 2) does not impact resistance to PS-3. PS-1, which is identical to PS-3 (Table 2) (Y = I) except for the triazole substituent (Table 2) (Y = H) showed >2.3-fold reduced activity against 3D7-C3^PS3^ versus that against 3D7-C3, suggesting that the iodo substituent (Y = I) of the triazole moiety is likely also not contributing to the resistance phenotype of 3D7-C3^PS3^ parasites. Overall, these structure-activity relationship (SAR) data implicate the glucose moiety in PS-3 as being associated with the resistance phenotype of 3D7-C3^PS3^ parasites.

**TABLE 2:** *In vitro* activity of PS glycoside analogues against P. falciparum 3D7-C3^PS3^ and 3D7-C3 parasites
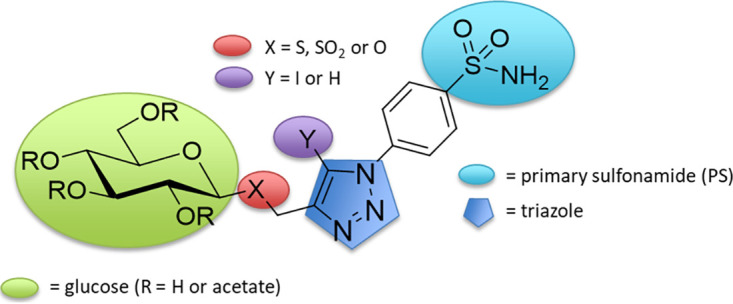

Compound	PS present	Structural feature^*a*^				IC_50_ (μM)^*b*^		Ri^*c*^	*P* value^*d*^
		Sugar	R	Y	X	3D7-C3	3D7-C3^PS3^		
PS-3	Yes	Glucose	Ac	I	S	1.25 (±0.64)	10.41 (±1.38)	8.3	0.0006
PS-3′	No	Glucose	Ac	I	S	6.42 (±1.43)	38.73 (±2.40)	6.0	0.0003
PS-7	Yes	Glucose	Ac	I	SO_2_	9.08 (±2.70)	65.53 (±4.90)	7.2	0.0351
PS-10	Yes	Glucose	Ac	I	O	5.15 (±1.42)	31.56 (±18.53)	6.1	0.1420
PS-11	Yes	Gal	Ac	I	S	5.03 (±2.50)	22.40 (±7.90)	4.5	0.0219
PS-12	Yes	GlcOMe	Ac	I	S	4.13 (±2.36)	19.62 (±6.95)	4.8	0.0217
PS-15	Yes	Mal	Ac	I	S	3.14 (±0.97)	7.83 (±0.85)	2.5	0.0032
PS-1	Yes	Glucose	Ac	H	S	43.19 (±9.04)	>100.00	>2.3	ND

a Gal, galactose; GlcOMe, glucuronic acid; Mal, maltose; Ac, acetate; H, hydrogen; I, iodine; S, sulfur; SO_2_, sulfur dioxide; O, oxygen.

b Mean IC_50_ ± SD for three independent experiments each in triplicate wells.

c Ri, resistance index: IC_50_ resistant line (3D7-C3^PS3^)/IC_50_ wild-type line (3D7-C3). The higher the Ri, the higher the level of resistance.

d Statistical difference between IC_50_s was determined using an unpaired *t* test with GraphPad Prism data analysis software.

To determine if sugars other than glucose play a role in the 3D7-C3^PS3^ resistance phenotype, PS-3 analogues differing only with respect to the sugar group were tested against 3D7-C3^PS3^ and 3D7-C3. 3D7-C3^PS3^ was found to be cross resistant to PS-11 (galactose replacing glucose) and PS-12 (glucuronic acid replacing glucose), as shown by similar Ri values to that for PS-3 (Table 2). To a lesser extent, 3D7-C3^PS3^ was found to be cross resistant to PS-14 (the disaccharide maltose replacing glucose; Ri, 2.5) (Table 2). Together, these SAR data suggest that the mode of action of PS analogues containing the sugars glucose, galactose, glucuronic acid, and, to a lesser extent, maltose may be affected by the phenotypic change associated with the 3D7-C3^PS3^ resistance. These sugars have subtle structural and stereochemical differences. Interestingly, 3D7-PfHK^+^, a parasite line that was previously shown to be resistant to the glucose analogue 2-deoxyglucose (14), is not resistant to PS-3 (data not shown).

### Whole-genome sequencing of 3D7-C3^PS3^ subclones identifies two independent single nucleotide polymorphisms in the P. falciparum phosphofructokinase (*Pf*PFK) gene.

**FIG 3:**
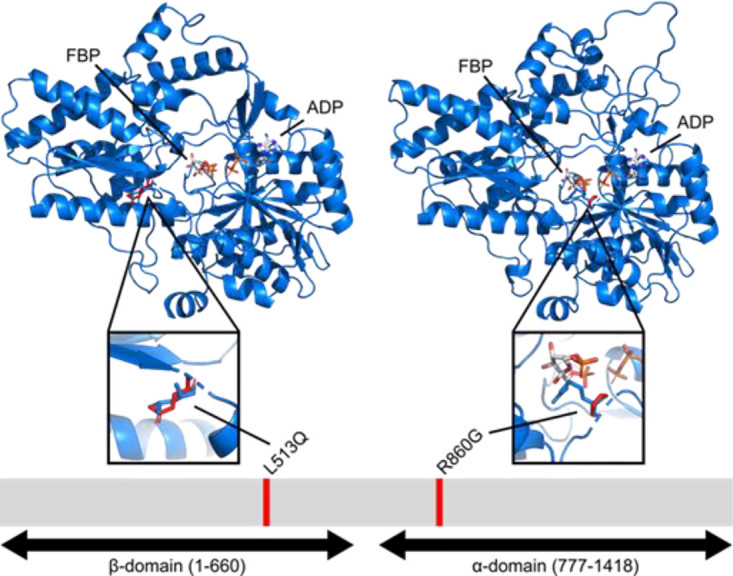
Mutations in 3D7-C3^PS3^ clones map to the β and α domains of *Pf*PFK9 (PF3D7_0915400). The glycoside-resistance allele L513Q (clone 3D7-C3^PS3-2^) maps to the β-domain and the R860G allele (clones 3D7-C3^PS3-1^ and 3D7-C3^PS3-3^) maps to the α-domain of *Pf*PFK9. (Top) Three-dimensional structural model of *Pf*PFK9, with wild-type residues in blue and predicted position of variant residues indicated in red. Products fructose 1,6-bisphosphate (FBP) and ADP (ADP) are shown as stick figures with a color scheme (carbon, white; nitrogen, blue; oxygen, red; phosphate, orange; hydrogen, not displayed). (Bottom) Domain structure of *Pf*PFK9, with positions of resistant variants indicated in red.

Three independent 3D7-C3^PS3^ subclones were generated (termed 3D7-C3^PS3-1^, 3D7-C3^PS3-2^, and 3D7-C3^PS3-3^), and their PS-3 resistance profiles were confirmed (Fig. 2C). The IC_50_ values of all the clones were significantly different from that of the wild-type 3D7-C3 parasites (Fig. 2C) (*P* < 0.05) but not for the control drug chloroquine (*P* > 0.05) (Fig. 2D). PS-3 resistance indices of 15.0, 6.6, and 10.8 were observed for 3D7-C3^PS3-1^, 3D7-C3^PS3-2^, and 3D7-C3^PS3-3^, respectively. A comparison of the genome sequences of the three clones versus that for 3D7-C3 revealed 12 new coding mutations, which included two point mutations (L513Q and R860G) in the *Pf*PFK9 gene (PF3D7_0915400) (Fig. 3; Table S1). The L513Q mutation was found only in clone 3D7-C3^PS3-2^ and maps to the β-domain of *Pf*PK9 (amino acids 1 to 660) (Fig. 3). The R860G mutation was found in clones 3D7-C3^PS3-1^ and 3D7-C3^PS3-3^ and maps to the α-domain (amino acids 777 to 1418) (Fig. 3). Mutations were observed in the P. falciparum multidrug-resistant protein 2 (*Pf*MDR2; *Pf*3D7_1447900) and the P. falciparum sodium/hydrogen exchanger (*Pf*NHE; *Pf*3D7_1303500) for the 3D7-C3^PS3-1^ and 3D7-C3^PS3-3^ clones (Table S1). The likelihood of finding three independent missense mutations in *Pf*PK9 by chance is very low (*P* = 1.7e−10, hypergeometric mean function). Furthermore, in contrast to that for *Pf*MDR*2*, no *Pf*PK9 mutations were detected in a systematic analysis of the genome sequences of 262 evolved parasites resistant to 37 diverse compounds (15) performed with the same sequence analysis pipeline. No other common gene amplifications were observed in the three independent clones analyzed.

10.1128/mBio.02842-20.10 TABLE S1 Resistance mutations found in annotated P. falciparum genes for 3D7-C3 and 3D7-C3^PS3^ clones. Download Table S1, XLSX file, 0.1 MB. Copyright © 2020 Fisher et al. 2020 Fisher et al. This content is distributed under the terms of the Creative Commons Attribution 4.0 International license.

### Modeling of glycoside resistance alleles.

To inform the possible structural basis of compound resistance, we evaluated the effects of resistance alleles (R860G and L513Q) on three-dimensional models of *Pf*PFK9 α and β domains (Fig. 3). Currently, no structure exists for the distinct “long” PFKs observed in apicomplexans. Our structural model shows that the R860G and L513Q mutations do not share cognate regions of their respective domains. The R860G mutation is adjacent to the substrate-binding pocket of the C-terminal α domain, while the L513Q mutation is buried between an alpha-helix and beta-strand of the N-terminal β domain and is not predicted to impact substrate-binding or catalytic residues. This suggests that these mutations may share their resistance phenotype through a more general mechanism of overall reduced PFK function rather than a specific change in a particular inhibitor-protein binding interface.

### *Pf*PFK9 R860G mutation validated as a resistance mechanism of PS-3.

CRISPR/Cas9-based genome editing was used to validate the contribution of the two *Pf*PFK9 mutations, L513Q and R860G, to the resistance phenotype. Donor templates were synthesized and cloned into a CRISPR vector encoding Cas9 and one of two single guide RNAs (sgRNAs) targeting the relevant locus (see Fig. S3). For each mutation, two donor templates were generated encoding either the putative resistance mutation (e.g., L513Q) or a silent control (e.g., L513L). Additional silent mutations common to both sets of donors were also included to prevent sgRNA binding to the repair locus (Fig. S3). The mutations were introduced into the Dd2 strain, and clonal lines were derived. The R860G-edited line demonstrated a 2.1-fold increase in IC_50_ values compared to that for the R860 silent control (*P* < 0.0001) (Fig. 2E). There was no significant change in IC_50_ values for the L513Q-edited line compared to that for the L513 silent control (*P* > 0.05) (Fig. 2F), which may possibly be due to the different genetic background (Dd2 versus 3D7) or the contribution of additional alleles. These data, nevertheless, provide evidence of an association between the *Pf*PFK9 R860G mutation and P. falciparum resistance to PS-3.

10.1128/mBio.02842-20.3 FIG S3 CRISPR/Cas9 genome editing of *Pf*PFK9. (A) Schematic of the Cas9-gRNA-donor plasmids for either the L513Q and L513 silent donor (left) or the R860G and R860 silent donor (right). (B) Genomic *Pf*PFK9 target site and the donor homology regions of 656 bp (L513Q) and 750 bp (R860G) of the synthesized donor templates. (C) Sequence of a region of the CRISPR donors, showing the gRNA binding sites and the desired mutations. Additional silent binding-site mutations (orange) were included in all donors to prevent gRNA binding and Cas9 cleavage of the edited genome. Download FIG S3, PDF file, 0.2 MB. Copyright © 2020 Fisher et al. 2020 Fisher et al. This content is distributed under the terms of the Creative Commons Attribution 4.0 International license.

### Mutations in *Pf*PFK9 lead to changes in central carbon metabolic flux.

**FIG 4:**
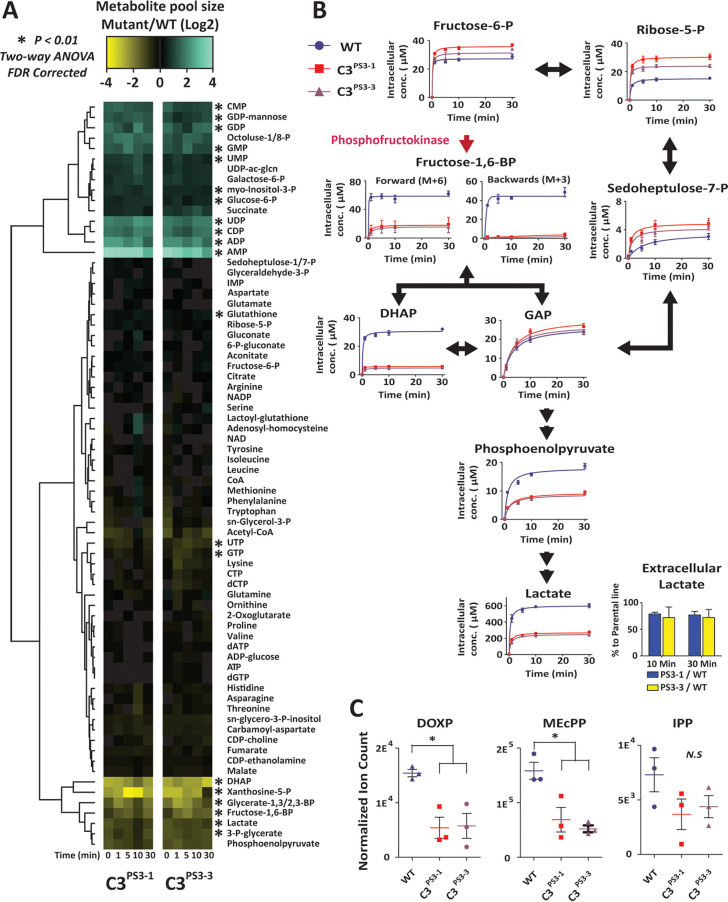
Dynamic ^13^C-U-glucose labeling of PS-3-sensitive and -resistant trophozoite-stage P. falciparum-infected erythrocytes. Purified (>95%) trophozoite-stage P. falciparum-infected erythrocytes were incubated in RPMI 1640 medium containing a 1:1 mixture of ^12^C/^13^C_6_-glucose (11 mM) and collected for LC-MS analysis across a 30-min time course. (A) Total metabolite pools were monitored over the time course and are expressed as the log_2_ ratio of the 3D7-C3^PS3-1^ or 3D7-C3^PS3-3^ to the wild-type parental strain. (B) The rate of ^13^C incorporation into glycolytic and pentose phosphate pathway intermediates. The *y* axis represents the amount of fully labeled metabolite as the estimated intracellular concentration (with the exception of fructose-1,6-Bisphosphate which depicts both the forward [M + 6], and backward [M + 3] species). The data presented in panels A and B represent the averages from three biological replicates (± standard errors of the means [SEMs]) and the excreted lactate data from two biological replicates (±SDs). (C) The intracellular pool of the isoprenoid biosynthetic intermediates in wild-type (WT) and 3D7-C3^PS3-1^ or 3D7-C3^PS3-3^ purified infected erythrocytes. The total pool sizes of deoxyribose-1-phosphate (DOXP), methylerythritol-cyclo-pyrophosphate (MEcPP), and isopentyl-pyrophosphate (IPP) are presented as arbitrary ion counts (normalized for data acquisition and degradation across different days), and represent three biological replicates performed on different days (means ± SEMs). One-way analysis of variance (ANOVA) testing was performed to test statistical significance. *, *P* < 0.05.

To investigate the impact of nonsynonymous mutations in *Pf*PFK on parasite metabolism, ^13^C-glucose labeling studies were undertaken on 3D7-C3^PS3-1^ and 3D7-C3^PS3-3^ (both containing the R860G mutation). Erythrocytes infected with trophozoite-stage parasites were labeled with ^13^C-glucose (present in medium at a 1:1 ratio with ^12^C-glucose) for 30 min, and incorporation into a wide range of intermediates in central carbon metabolism was quantitated by liquid chromatography-mass spectrometry (LC-MS). The pool sizes of several glycolytic intermediates immediately downstream of *Pf*PFK, including fructose-1,6-bisphosphate and dihydroxyacetone phosphate (DHAP), were significantly reduced in the mutant lines, whereas those of several nucleoside mono- and diphosphates were elevated (Fig. 4A). The level of ^13^C enrichment in these downstream intermediates was also greatly reduced, suggesting that the metabolic flux through PFK was severely compromised in 3D7-C3^PS3-1^- and 3D7-C3^PS3-3^-infected erythrocytes (Fig. 4B). Reduced flux through the reversible aldolase reaction was further supported by the markedly reduced levels of +3-labeled fructose-1,6-bisphosphate in the mutant lines, which reflects the rate of interconversion of DHAP/glyceraldehyde-3-phosphate (GAP) and fructose-1,6-bisphosphate. These labeling studies indicate that mutations acquired in 3D7-C3^PS3-1^ and 3D7-C3^PS3-3^ parasites lead to reduced *Pf*PFK activity. Interestingly, ^13^C enrichment in GAP, an intermediate in both glycolysis and the pentose phosphate pathway (PPP), was unaltered in 3D7-C3^PS3-1^ and 3D7-C3^PS3-3^ parasites, while labeling of several intermediates in the oxidative and nonoxidative PPP (sedoheptulose-7-P and ribose-5-P) was increased in these mutant lines. These data strongly suggest that partial loss of *Pf*PFK activity leads to rerouting of carbon flux through the PPP, leading to the production of GAP that can still be catabolized in lower glycolysis. This bypass would allow ATP-producing steps in lower glycolysis to proceed, albeit at a reduced rate, as evidenced by the lower levels of synthesis of phosphoenolpyruvate (PEP) and lactate (Fig. 4B). The reduction of glycolytic flux in 3D7-C3^PS3-1^ and 3D7-C3^PS3-3^ was consistent with an approximate 25% reduction in extracellular lactate secretion (Fig. 4B). The rerouting of carbon flux through the PPP, at the expense of glycolysis, would be expected to come with a fitness disadvantage as a result of reduced ATP production. This hypothesis was supported by the significant increase in ADP and AMP levels in the mutant lines, leading to reduced ATP/ADP and ATP/AMP ratios (Fig. 4A). Glycolysis is also the source of precursors for a number of anabolic pathways, such as isoprenoid biosynthesis, which uses phosphoenolpyruvate and DHAP generated downstream of PFK. Lastly, the isoprenoid biosynthetic intermediates deoxyribose-1-phosphate (DOXP) and methylerythritol-cyclo-pyrophosphate (MEcPP) were significantly reduced in both 3D7-C3^PS3-1^ and 3D7-C3^PS3-3^, indicating reduced flux into this pathway and an additional fitness disadvantage to PFK mutations (Fig. 4C).

### 3D7-C3^PS3^ subclones display slowed *in vitro* growth compared to that of wild-type 3D7-C3 parasites.

To assess *in vitro* growth dynamics of PS-3-resistant parasites, the *in vitro* growth profiles of 3D7-C3^PS3-1^ and 3D7-C3^PS3-3^, compared to that of wild-type 3D7-C3 parasites, were examined by comparing growth over 72 h commencing at ∼3 to 6 h postinvasion (see Fig. S4). No significant difference in parasitemia was seen up to 30 h postinvasion for 3D7-C3^PS3-1^ and 3D7-C3^PS3-3^ compared to that for 3D7-C3. In contrast, at 51 to 54 h and 75 to 78 h postinvasion, a >2-fold reduction in total parasitemia was observed for both 3D7-C3^PS3^ subclones compared to that of the wild type (*P* < 0.05 and *P* < 0.01, respectively) (Fig. S4). Upon examination of each developmental stage, no significant difference was seen in the percentages of each stage (early rings, mid/late rings, early/mid trophozoites, late trophozoites/early schizonts, and late schizonts) up to 30 h postinvasion for 3D7-C3 versus those for 3D7-C3^PS3-1^ and 3D7-C3^PS3-3^ (*P* > 0.05) (see Fig. S5A and B). However, at 51 to 54 h postinvasion, 2-fold reductions were seen in the percentages of rings for 3D7-C3^PS3-1^ and early/mid trophozoites for 3D7-C3^PS3-3^ compared to those for 3D7-C3 (*P* < 0.05) (Fig. S5C). Additionally, at 72 to 78 h postinvasion, a 2-fold increase in the percentage of late trophozoite/early schizonts and a 5-fold decrease in the percentage of late schizonts was observed for both 3D7-C3^PS3^ subclones compared to those for the wild type (*P* < 0.01 and *P* < 0.05, respectively) (Fig. S5D). Overall, these data suggest that parasite growth is developmentally delayed in 3D7-C3^PS3-1^ and 3D7-C3^PS3-3^ parasites.

10.1128/mBio.02842-20.4 FIG S4 *In vitro* growth analysis of P. falciparum 3D7-C3^PS3^ clones versus 3D7-C3 wild-type parasites. Growth of 3D7-C3^PS3-1^, 3D7-C3^PS3-3^, and 3D7-C3 P. falciparum parasites over 72 h (starting at ∼3 to 6 h postinvasion) was determined by microscopic examination of Quickdip-stained thin blood smears taken every 24 h. Mean number of parasites per 100 red blood cells (RBCs) was determined at each time point by examining >3,000 infected RBCs by two independent microscopists. Results are means (±SDs) from three independent experiments. *P < 0.05; **P < 0.01. Download FIG S4, PDF file, 0.2 MB. Copyright © 2020 Fisher et al. 2020 Fisher et al. This content is distributed under the terms of the Creative Commons Attribution 4.0 International license.

10.1128/mBio.02842-20.5 FIG S5 *In vitro* developmental stage analysis of asexual intraerythrocytic P. falciparum 3D7-C3^PS3^ clones versus 3D7-C3 wild-type parasites. Different asexual intraerythrocytic developmental stages of 3D7-C3^PS3-1^, 3D7-C3^PS3-3^, and 3D7-C3 P. falciparum parasites were assessed at 3 to 6 h (A), 27 to 30 h (B), 51 to 54 h (C), and 75 to 78 h (D) postinvasion (>150 parasites counted per time point). Data are the means (±SDs) of each developmental form as a percentage of the total number of parasites for three independent assays. ER, early ring; M/LR, mid to late ring; E/MT, early to mid-trophozoite; LT/ES, late trophozoite to early schizont; LS, late schizont. *P < 0.05; **P < 0.01. Download FIG S5, PDF file, 0.2 MB. Copyright © 2020 Fisher et al. 2020 Fisher et al. This content is distributed under the terms of the Creative Commons Attribution 4.0 International license.

### Metabolite profiling of 3D7-infected red blood cells treated with PS-3.

**FIG 5:**
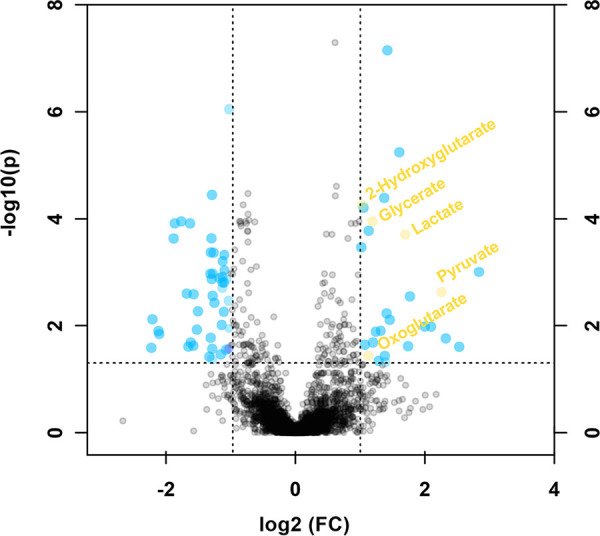
Metabolite perturbations following exposure to PS-3. Untargeted metabolite profiling of purified trophozoite-stage parasite-infected erythrocytes treated with 40 μM PS-3 (2 h). Mass-to-charge (*m/z*) features were aligned and a pairwise comparison performed between PS-3-treated and untreated purified infected erythrocytes using a *P* value of <0.05 (FDR corrected) and fold change of 2 cutoffs for determining significant peaks. Data are presented as the mean ratios from four independent replicates. Putative metabolite identifications were made via the METLIN database and confirmed with authentic standards (highlighted yellow).

To investigate whether *Pf*PFK is a direct target of PS-3 or if mutations in *Pf*PFK provide a metabolic bypass that indirectly confers resistance, erythrocytes infected with purified trophozoite-stage P. falciparum 3D7 were treated with PS-3 (40 μM; equivalent to 4× IC_50_ for this parasite life cycle stage at 48 h of exposure) for 2 h, and changes in metabolite levels determined using untargeted LC-MS profiling (Fig. 5). More than 90 of the 3,327 detected mass-to-charge (*m/z*) features were significantly different (*P* < 0.05, false-discovery rate [FDR] corrected) between those treated with PS-3 and the untreated control. The METLIN database was queried for possible identities using the M-H criteria with a mass tolerance of 10 ppm (assuming that metabolites existing as other adducts will also be present as the M-H adduct). Several features corresponded to glycolytic intermediates, and these were subsequently verified using authentic standards (Fig. 5, highlighted in yellow). In particular, lactate and pyruvate were significantly increased following PS-3 treatment, with glycerate and the tricarboxylic acid (TCA) cycle-linked intermediates 2-oxoglutarate and 2-hydroxyglutarate also significantly elevated. This metabolic phenotype is distinct from that observed in the PS-3-resistant lines, indicating that PS-3 does not directly target PFK but inhibits other steps in central carbon metabolism that are compensated for by rerouting of glucose into the pentose phosphate pathway and/or reduced flux in lower glycolysis.

### Antiplasmodial glycosides do not directly inhibit apicomplexan phosphofructokinases.

**FIG 6:**
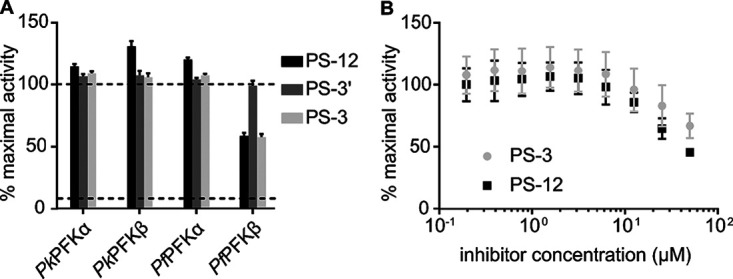
Antiplasmodial glycosides do not inhibit *Plasmodium* phosphofructokinases. (A) Compounds PS-3, PS-3′, and PS-12 were screened for activity (at 50 μM) against recombinant protein domains *Pk*PFKα, *Pk*PFKβ, *Pf*PFKα, and *Pf*PFKβ; dotted lines represent maximal activity for each enzyme and minimal activity from assay background as determined from a catalytically dead *Pf*PFK*β*. (B) Inhibitory dose response of *Pf*PFKβ for compounds PS-3 and PS-12.

To further evaluate whether *Pf*PFK9 represents the direct target of antiplasmodial glycosides, we evaluated compound sensitivity against P. falciparum and Plasmodium knowlesi PFK9. While we, and others, have not been able to purify full-length *Pf*PFK9 (5), P. falciparum and P. knowlesi α/β domains readily express as active individual domains (see Fig. S6). We screened each recombinant protein for enzymatic inhibition at a single compound concentration (50 μM) (Fig. 6A), revealing possible modest inhibition of purified *Pf*PFKβ by compounds PS-3 and PS-12. However, dose-responsive inhibition of *Pf*PFKβ confirmed little to no enzymatic inhibition by these compounds at concentrations similar to the compound’s cellular inhibition values (biochemical IC_50_s as follows: PS-3, >22 ± 4.33 μM; PS-12, >21 ± 1.49 μM) (Fig. 6B). The lack of direct inhibition of any domain of these *Pf*PFK enzymes suggest *Pf*PFK9 may not be the direct target of PS-3.

10.1128/mBio.02842-20.6 FIG S6 Coomassie gel images of purified PFK orthologs. (Left) Heterologous protein expression of Plasmodium knowlesi and Plasmodium falciparum alpha and beta subunits (designated by red arrows). Protein identity was confirmed by expected band size and mass spectrometry. (Right) *Pf*PFK subunits following additional purification using size exclusion chromatography. Download FIG S6, PDF file, 0.3 MB. Copyright © 2020 Fisher et al. 2020 Fisher et al. This content is distributed under the terms of the Creative Commons Attribution 4.0 International license.

### *Pf*PFK mutants are not resistant to *Plasmodium* lactate transporter inhibitors.

The increases observed in the lower glycolytic intermediates lactate, pyruvate, and glycerate in PS-3-treated trophozoites raised the possibility that PS-3 may act by blocking lactate efflux across the parasite plasma membrane. The *Plasmodium* formate nitrite transporter (*Pf*FNT) mediates the efflux of lactate from the malaria parasite (16, 17), and two malaria box compounds (MMV007839 and MMV000972) were recently shown to inhibit lactate transport via inhibition of *Pf*FNT (18). To further investigate if PS-3 targeting is related to *Pf*FNT inhibition, the *Pf*PFK R860G and L513Q mutant parasites were screened against MMV007839 and MMV000972 in 72-h growth inhibition assays. No significant difference (*P* > 0.05) was observed in IC_50_ values for each compound against both the mutant and silent control lines (MMV 007839: R860G IC_50_, 0.13 ± 0.01 μM; R860 silent IC_50_, 0.15 ± 0.02 μM; MMV 000972: R860G IC_50_, 0.76 ± 0.08 μM; R860 silent IC_50_, 0.81 ± 0.12 μM; MMV 007839: L513Q IC_50_, 0.13 ± 0.06 μM; L513 silent IC_50_, 0.15 ± 0.03 μM; MMV 000972: L513Q IC_50_, 0.59 ± 0.27 μM; L513 silent IC_50_, 0.93 ± 0.16 μM) (see Fig. S7). This lack of cross-resistance suggests that PS-3 may not target *Pf*FNT.

10.1128/mBio.02842-20.7 FIG S7 *In vitro* profile of P. falciparum *Pf*PFK9 mutant parasites against *Pf*FNT inhibitors. The mean percent growth inhibition (±SD) of P. falciparum PfPFK9 mutant lines and controls R860G/R860 silent (A and B) and L513Q/L513 silent (C and D) against the PfFNT inhibitors MMV007839 (A and C) and MMV000972 (B and D) was assessed using 72-h [^3^H]hypoxanthine uptake growth inhibition assays. In each case, four independent assays, each in triplicate wells, were carried out, and mean (±SD) 50% inhibitory concentrations (IC_50s_) were determined using nonlinear regression analysis in GraphPad prism. Download FIG S7, PDF file, 0.2 MB. Copyright © 2020 Fisher et al. 2020 Fisher et al. This content is distributed under the terms of the Creative Commons Attribution 4.0 International license.

### PS-3-resistant clones are hypersensitive to fosmidomycin.

**FIG 7:**
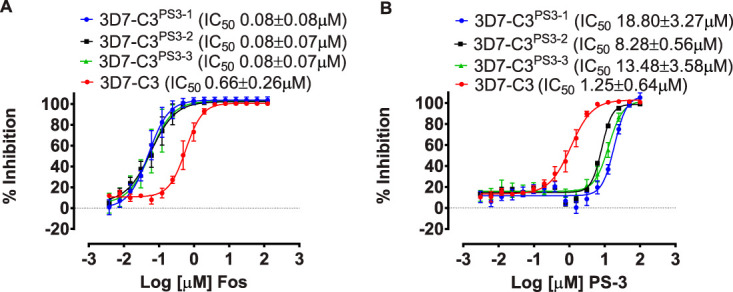
*In vitro* profile of P. falciparum PS-3-resistant clones against fosmidomycin. The sensitivity of P. falciparum 3D7-C3^PS3^ subclones 3D7-C3^PS3-1^, 3D7-C3^PS3-2^, 3D7-C3^PS3-3^, and 3D7-C3 against compound fosmidomycin (A) and the control compound PS-3 (B) was assessed using 72-h [^3^H]hypoxanthine uptake growth inhibition assays. In each case, ≥3 independent assays, each in triplicate wells, were carried out, and 50% inhibitory concentrations (IC_50_) were determined using nonlinear regression analysis in GraphPad prism.

Our metabolomic studies indicated that glycolytic flux is reduced in 3D7-C3^PS3^ subclones, which leads to decreased flux of glycolytic intermediates into the apicoplast isoprenoid DOXP biosynthetic pathway (Fig. 4C). Previous studies on the *Pf*HAD1 mutants have shown that changes in glycolytic flux directly impact the resistance of asexual intraerythrocytic-stage P. falciparum to fosmidomycin (9), a potent competitive inhibitor of the apicoplast MEP pathway enzyme deoxyxyluose phosphate reductoisomerase (*Pf*DXR) (19). We therefore assessed whether the observed decrease in glycolytic flux in the PS-3-resistant lines was associated with increased sensitivity to fosmidomycin in 72-h growth inhibition assays. A significant increase in activity for fosmidomycin was seen for all three 3D7-C3^PS3^ clones compared to that for the 3D7-C3 wild-type parasites (Fig. 7A) (∼8-fold higher IC_50_ values; *P* < 0.01 [3D7-C3^PS3-1^ and 3D7-C3^PS3-2^], *P* < 0.05 [3D7-C3^PS3-2^]). As previously observed (Fig. 2C), PS-3 showed a significant decrease in activity for all three 3D7-C3^PS3^ clones compared to the that for 3D7-C3 (Fig. 7B) (∼7- to 15-fold lower IC_50_s; *P* < 0.001). The PS-3-resistant parental line (3D7-C3^PS3^) also displayed hypersensitivity to the P. falciparum apicoplast inhibitor clindamycin (see Fig. S8) (∼3-fold higher IC_50_ values for 3D7-C3 compared to that for 3D7-C3^PS3^; *n* = 2). Collectively, these data support the hypothesis that resistance to PS-3 in the 3D7-C3^PS3^ clones is associated with metabolic adaptation that leads to decreased availability of glycolytic intermediates for anabolic pathways, such as isoprenoid biosynthesis.

10.1128/mBio.02842-20.8 FIG S8 *In vitro* profile of P. falciparum PS-3-resistant parasites against clindamycin. The mean percent growth inhibition (±SD) of P. falciparum 3D7-C3^PS3^ and 3D7-C3 parasites against clindamycin (A) and the control compound PS-3 (B) was assessed using 96-h [^3^H]hypoxanthine uptake growth inhibition assays. In each case, 2 independent assays, each in triplicate wells, were carried out, and mean (±SD) 50% inhibitory concentrations (IC_50s_) were determined using nonlinear regression analysis in GraphPad prism. Download FIG S8, PDF file, 0.2 MB. Copyright © 2020 Fisher et al. 2020 Fisher et al. This content is distributed under the terms of the Creative Commons Attribution 4.0 International license.

### Increased glycolytic flux may also result in resistance to PS-3.

**FIG 8:**
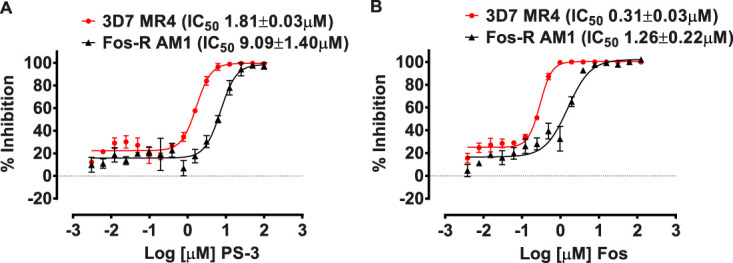
*In vitro* profile of P. falciparum fosmidomycin-resistant parasites against PS-3. The sensitivity of P. falciparum Fos^r^ 3D7-AM1 and wild-type 3D7-MR4 against compound PS-3 (A) and fosmidomycin (B) was assessed using 72-h [^3^H]hypoxanthine uptake growth inhibition assays. In each case, three independent assays, each in triplicate wells, were carried out, and 50% inhibitory concentrations (IC_50_) were determined using nonlinear regression analysis in GraphPad prism.

**FIG 9:**
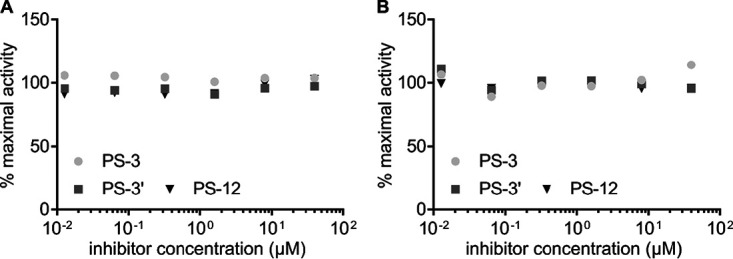
Antimalarial glycosides do not inhibit recombinant *Pf*HAD1 and *Pf*HAD2. Inhibitory dose-responses for antimalarial glycosides against recombinant proteins *Pf*HAD1 (A) and *Pf*HAD2 (B).

To further investigate the mode of action of PS-3 and potential resistance mechanisms, we investigated the sensitivity of the P. falciparum *had1* mutant, AM1, to PS-3. The *Pfhad1* gene encodes a haloacid dehalogenase that dephosphorylates a range of glycolytic intermediates *in vitro* and appears to be involved in negatively regulating glycolytic flux. Loss-of-function mutations in *Pfhad1* are associated with increased flux of glycolytic intermediates into isoprenoid synthesis and resistance to fosmidomycin (9). The AM1 parasite line exhibited a 5-fold increase in resistance to PS-3 compared to that of the parental 3D7 line (*P* < 0.001) (Fig. 8A). This level of resistance is comparable to that exhibited by the AM1 line to fosmidomycin (i.e., 4-fold increase in IC_50_; *P* < 0.05) (Fig. 8B). This finding suggests that global changes in central carbon metabolism that can be associated with either an increase in glycolytic flux (as occurs in AM1) or a decrease in glycolytic flux (as occurs in the 3D7-C3^PS3^ subclones) increase the resistance of asexual stages to PS-3. However, unlike the control fosmidomycin, the antiplasmodial activity of PS-3 was not rescued by supplementation with isoprenoid isopentenyl pyrophosphate (IPP) (see Fig. S9). Furthermore, we find that PS-3 does not directly inhibit *Pf*HAD1 or *Pf*HAD2 enzyme activity *in vitro* (Fig. 9). Together, these data strongly show that PS-3 has a distinct mechanism of action from other direct MEP pathway inhibitors, such as fosmidomycin.

10.1128/mBio.02842-20.9 FIG S9 PS-3 *in vitro* activity is not rescued by IPP supplementation. The sensitivity of P. falciparum 3D7 against compound PS-3 (A) and fosmidomycin (B) without IPP (red lines) and with 200 μM IPP (black lines) was assessed using 72-h [^3^H]hypoxanthine uptake growth inhibition assays. In each case, the mean percent inhibition (±SD) compared to that of DMSO controls was determined for three independent assays, each carried out in triplicate wells. Mean (±SD) 50% inhibitory concentrations (IC_50s_) were determined using nonlinear regression analysis in GraphPad prism. Download FIG S9, PDF file, 0.4 MB. Copyright © 2020 Fisher et al. 2020 Fisher et al. This content is distributed under the terms of the Creative Commons Attribution 4.0 International license.

## DISCUSSION

Malaria remains a major global concern with efforts to control this disease being hampered by parasite drug resistance and the lack of a broadly effective vaccine (1). Although there are several new chemotherapeutics in various stages of preclinical or clinical development, most of these have previously exploited targets or are reformulations or different combinations of existing antimalarial drugs (20). Therefore, strategies to identify new antimalarial drug targets and antiplasmodial chemotypes that have novel modes of action are crucial. Fortunately, recent reductions in the cost of whole-genome sequencing and the development of comprehensive global metabolomic profiling approaches have begun to yield some exciting progress in the antimalarial target identification arena. When combined with the selection of drug-resistant P. falciparum lines, this approach has resulted in the identification of new antimalarial targets and resistance mechanisms (11, 21, 22).

In this study, *in vitro* resistance selection was utilized to generate P. falciparum parasites resistant to an antiplasmodial glycoside (PS-3) (10). Importantly, PS-3-resistant parasites displayed no *in vitro* resistance to several clinically used antimalarial drugs or to two compounds that represent chemical classes under clinical development (11, 12), suggesting a novel mode of action/resistance. Historically, the PS moiety is known to inhibit carbonic anhydrase (CA) enzyme activity in many organisms (23), including P. falciparum (24, 25). In this study, we demonstrate that PS-3 resistance in P. falciparum is not linked to the PS moiety, as no change in the resistance profile was observed for PS-3′, the non-PS structural analogue of PS-3 (Table 2). Further screening of PS glycoside analogues against PS-3-resistant parasites revealed that the glucose component of PS-3 may be contributing to the resistance phenotype. These data support genome sequencing data of 3D7-C3^PS3^ clones and subsequent validation using reverse genetics, which indicate that the point mutation (R860G) in *PfPFK*9, the gene that encodes an isoform of the glycolytic enzyme phosphofructokinase, contributes to PS-3 resistance. However, the resistance index of the R860G-edited line was lower than that observed for the 3D7-C3^PS3-1^ and 3D7-C3^PS3-3^ clones (Ri, ∼2.1, 10.8, and 15, respectively). This may be attributed to mutations in the P. falciparum multidrug-resistant protein 2 (*Pf*MDR2; *Pf*3D7_1447900) and/or the P. falciparum sodium/hydrogen exchanger (*Pf*NHE; *Pf*3D7_1303500), observed in the 3D7-C3^PS3-1^ and 3D7-C3^PS3-3^ clones only (see Table S1 in the supplemental material). The role of *Pf*MDR2 in antimalarial drug resistance is unclear, with some weak evidence linking resistance to pyrimethamine (26) and sulfadoxine (27) treatments. Likewise, there are contradictory reports on the association of sequence polymorphisms in the *Pf*NHE gene and quinine resistance (28–31). In this study, the 3D7-C3^PS3^ parasites were not resistant to pyrimethamine and quinine (Table 1); however, the *Pf*MDR2 and *Pf*NHE mutations observed in the 3D7-C3^PS3-1^ and 3D7-C3^PS3-3^ clones were not reported previously; therefore, further studies are required to confirm if these mutations are associated with resistance to PS-3. While mutations in *Pf*MDR2 and *Pf*NHE genes may be contributing to PS-3 resistance, this is more likely to be via nonspecific multidrug resistance mechanisms (32, 33). On the other hand, the PFK enzyme has an essential housekeeping role in P. falciparum central carbon metabolism; therefore, mutations in this gene are more likely to be target associated. Surprisingly, the L513Q-edited line was not resistant to PS-3, and the 3D7-C3^PS3-2^ clone containing this mutation did not share any other common mutations with the other two clones (Table S1). In this case, it is possible that multiple background mutations (Table S1) are contributing to the resistance profile of the 3D7-C3^PS3-2^ clone and masking the effect that the L513Q mutation may be exhibiting. Alternatively, our modeling suggests that the L513Q mutation may have less of an effect on *Pf*PFK function due to its more distal proximity to the substrate-binding pocket than the R860G mutation (Fig. 3). It should also be noted that the L513Q clone reported in this study is a subclone of the original L513Q clone generated. This original clone did display resistance to PS-3 (∼6-fold increase in IC_50_ [*n* = 1] for two independent PS-3 stocks; data not shown). However, this phenotype was lost following cryopreservation, and a reversion to the wild-type genotype was observed. It is therefore conceivable that the L513Q subclone may have gained mutations in another region of the genome that may have restored sensitivity to PS-3. Nonetheless, overall, these data strongly suggest that the *Pf*PFK R860G mutation contributes to PS-3 resistance, suggesting that alterations in glycolytic flux help to bypass the mode of action of PS-3.

**FIG 10:**
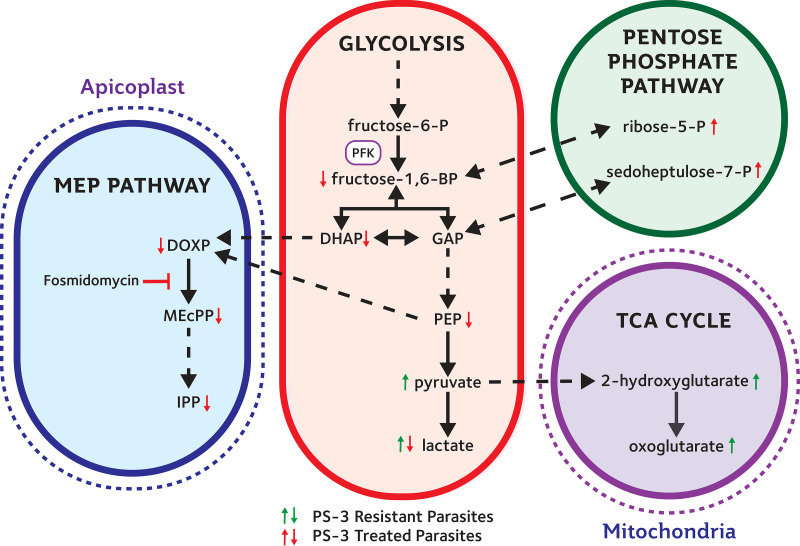
Overview of the central carbon pathways and metabolites associated with PS-3 activity and resistance. Upregulation (↑) and downregulation (↓) of metabolites observed in PS-3-resistant parasites (red arrows, ^13^C glucose labeling studies) and PS-3-treated parasites (green arrows, metabolic profiling of PS-3 treated *Pf*3D7). PFK, phosphofructokinase. DHAP, dihydroxyacetone phosphate; GAP, glyceraldehyde-3-phosphate; PEP, phosphoenolpyruvate; DOXP, deoxyribose-1-phosphate; MEcPP, methylerythritol-cyclo-pyrophosphate; IPP, isopentenyl-pyrophosphate.

To further understand the impact that the R860G *Pf*PFK mutation has on P. falciparum metabolism, ^13^C-glucose fluxes were measured in both the PS-3-resistant clones (3D7-C3^PS3-1^ and 3D7-C3^PS3-3^) and 3D7-C3 wild-type parasites. These studies revealed a profound rewiring of glucose fluxes in the two resistant clones. In particular, a reduction in labeling of intermediates immediately downstream of PFK was associated with increased flux of ^13^C-glucose into the PPP (Fig. 4). The redirection of glucose-6-phosphate into the PPP likely accounts for the reduction of ATP/ADP and ATP/AMP ratios and reduced *in vitro* growth of PS-3-resistant parasites (Fig. S4). On the other hand, increased flux into the PPP would allow regeneration of NADPH and contribute to a more robust redox state. Surprisingly, our data suggest that *Pf*PFK may not be the direct target of PS-3, as PS-3 was unable to inhibit recombinant *Pf*PFK and P. knowlesi PFK (*Pk*PFK) enzymes (Fig. 6). Furthermore, *in silico* homology modeling predicts that PS-3 is not likely to affect PFK substrate binding or catalysis. Nonetheless, it should be noted that PS-3 was only tested against the individual subunits of *Pf*PFK, as no full-length enzyme is available; thus, we cannot completely rule out *Pf*PFK9 as a target of PS-3. However, PS-3 could in principal inhibit another enzyme in the glycolytic pathway or in the pentose phosphate pathway. Initially, we hypothesized that PS-3 may inhibit the parasite’s lactate transporter (P. falciparum formate nitrite transporter, or *Pf*FNT, a validated drug target [16, 17]), resulting in a toxic buildup of metabolites. This would be consistent with the increases seen in the lower glycolytic intermediates (lactate, pyruvate, and glycerate) in PS-3-treated trophozoites (Fig. 10). PS-3-resistant parasites may overcome this toxicity by redirecting glycolytic flux away from lower glycolysis, resulting in less lactate being excreted and hence reducing the potency of PS-3. However, the L513Q and R860G mutant parasites were not cross resistant to the *Pf*FNT inhibitors MMV000972 and MMV007839 (18) (Fig. S7). Moreover, the elevation of pyruvate and lactate following PS-3 treatment is consistent with *Pf*FNT inhibition, but PS-3 treatment does not lead to the additional perturbations reported for *Pf*FNT inhibition (e.g., pyrimidine biosynthesis and hemoglobin catabolism [18]). However, it is possible that differences in lactate accumulation may exist between *Pf*3D7 and *Pf*Dd2, warranting *Pf*FNT inhibition studies against the 3D7 PS-3-selected clones. To the best of our knowledge, there are no reports comparing 3D7 and Dd2 lactate metabolism, but we do know that 3D7 lactate metabolic profiles are similar to the profiles of other multidrug-resistant strains 7G8 and K1 (34) and a *Pf*3D7 chloroquine-resistant transporter (*Pf*CRT) mutant line (35). Therefore, while we cannot completely discount the possibility that PS-3 may partially block *Pf*FNT, with the evidence presented, we think another target is leading to the observed disruptions and eventual cell death. For example, PS-3 may inhibit an enzyme in the PPP, accounting for the accumulation of intermediates in glycolysis (glucose-6-P, pyruvate, lactate) and the TCA cycle (2-oxoglutarate and 2-hydroxyglutarate). In this case, PS-3-resistant parasites may have adapted by diverting the glycolytic intermediates (fructose-6-phosphate [F6P] and GAP) into the nonoxidative arm of the pentose phosphate pathway to overcome the effects of PS-3 (Fig. 10). In P. falciparum, the nonoxidative reactions of the PPP are mainly designed to produce ribose-5-phosphate (R5P) for nucleic acid synthesis. The nonoxidative arm of the PPP can also utilize the glycolytic intermediates (F6P and GAP) to produce R5P and vice versa (36). Two key enzymes involved in these processes are glucose-6-phosphate dehydrogenase 6-phosphogluconolactonase (*Pf*GluPho) (37) and transketolase (38), both of which have been characterized, are essential for asexual growth (6, 37), and differ structurally from their human homologues (37, 38), making them potential drug targets. Finally, it is possible that PS-3 may target other processes that lead to increased oxidative stress or loss of redox balance, such as mitochondrial metabolism, which is compensated for by increased flux through the PPP (with concomitant regeneration of NADPH).

In addition to reduced glycolytic flux, we also observed a reduction in the MEP pathway metabolites (DOXP, MEcPP, and IPP) in PS-3-resistant parasites (Fig. 10). The MEP pathway is essential for the synthesis of isoprenoids in P. falciparum (39) and is dependent on the continuous import of glycolytic intermediates from the cytoplasm (9). The lead antimalarial drug fosmidomycin targets the MEP pathway enzyme, deoxyxylulose phosphate reductoisomerase (DXR), and fosmidomycin resistance has been linked to mutations in P. falciparum haloacid dehalogenases (*Pf*HAD1/2) (7, 9). While we found that fosmidomycin-resistant parasites (Fos^r^ AM1 *Pf*HAD1 mutant) were also resistant to PS-3, the activity of PS-3 was not able to be rescued by IPP (isoprenoid precursor), and PS-3 did not inhibit *Plasmodium* HAD1/HAD2 recombinant enzymes. This suggests that PS-3 has a different mechanism of action/resistance to fosmidomycin. In contrast, PS-3-resistant parasites were found to be hypersensitive to fosmidomycin. Given that fosmidomycin targets DXR, it is likely that the reduction in DXR’s substrate (DOXP) in PS-3-resistant parasites, coupled with a decrease in MEcPP and IPP, results in an increase in fosmidomycin activity. Increased sensitivity to fosmidomycin has also been reported in P. falciparum parasites lacking phosphoglycolate phosphatase (PGP) (40). PGP, a third member of the P. falciparum HAD family, has been shown to be involved in regulating glycolysis and PPP flux in asexual P. falciparum (40). In Δ*pgp* parasites, the loss of PGP leads to the inhibition of the PPP enzyme 6-phosphogluconate dehydrogenase (6-PGD), resulting in reduced glycolytic flux and causing reduced isoprenoid biosynthesis and increased sensitivity to fosmidomycin (40). Interestingly, fosmidomycin-resistant parasites harboring mutations in *Pf*HAD2 display the opposite metabolomic profile to that of PS-3-resistant parasites. As mentioned above, PS-3-resistant parasites exhibit decreased levels of the metabolites FBP, DOXP, and MEcPP, whereas a reported increase in these metabolites is seen in *Pf*HAD2 mutants (7). In *Pf*HAD2 mutants, the increase in FBP is thought to lead to the increase of glycolytic intermediates into the MEP pathway, which results in an increase of MEP pathway metabolites, thus reducing the sensitivity to fosmidomycin (7). Furthermore, mutations in *Pf*PFK9 have been shown to restore fosmidomycin sensitivity in these *Pf*HAD2 mutant parasites (7); however, these mutations are not the same as those seen in PS-3-resistant parasites. Overall, these findings provide further evidence that *Pf*PFK9 plays an important regulatory role in central carbon metabolism to modify drug sensitivity.

In this study, we identified a novel mechanism of P. falciparum drug resistance and have further highlighted the importance of the role of *Pf*PFK9 in regulating central carbon metabolism to offset the effects of antiplasmodial compounds. While the specific molecular target of PS-3 remains unclear, it is promising that no cross-resistance exists with several of our clinically used antimalarials, indicating that this chemical class is likely to have a different mode of action from that of our current drugs. Furthermore, our PS-3-resistant parasites will provide a useful tool for studying central carbon metabolism in P. falciparum and aid in identifying other inhibitors of these pathways for development as antimalarial drug leads.

## MATERIALS AND METHODS

### Compounds.

PS glycosides and matched control compounds were synthesized as previously reported by us (10). Chloroquine, artesunate, pyrimethamine, and cytochalasin B were purchased from Sigma-Aldrich, USA. DSM161 (12) was supplied by Margaret Philips from UT Southwestern, Dallas, TX, USA. Stock solutions were prepared in 100% dimethyl sulfoxide (DMSO; Sigma-Aldrich, USA), stored at −20°C and diluted as required.

### P. falciparum *in vitro* culture.

P. falciparum-infected erythrocytes were cultured in O-positive human erythrocytes in RPMI 1640 medium (Gibco, USA) containing 10% heat-inactivated pooled human sera and 5 μg/ml gentamicin (Sigma, USA). Cultures were maintained at 37°C in a gas mixture composed of 5% O_2_, 5% CO_2_, and 90% N_2_, as described previously (41).

### Cloning of P. falciparum parasites by limiting dilution.

P. falciparum-infected erythrocytes were cultured under standard culture conditions and synchronized to ring stage using sorbitol treatment (42). Synchronous ring-stage parasites were cultured for two cycles (96 h) on a plate shaker (Gyro mini; Labnet, USA) under standard culture conditions until multiple infections were less than 1%, as determined by microscopic examination of Quickdip-stained (POCD, Australia) thin blood films. Following cell counts with a hemocytometer, cultures were diluted to 0.5 and 0.1 parasites per 200 μl (2% hematocrit), dispensed into sterile 96-well plates (Corning, USA), and incubated under standard culture conditions with medium replaced weekly. On day 16, stained thin blood smears were prepared and stained with Quickdip (POCD) and then examined by microscopy. Parasite-positive cultures were transferred into 50-mm petri dishes (Corning) and then 100-mm petri dishes (Corning) for expansion and cryopreservation. The parasite-negative wells were checked 1 week later by microscopic analysis of Quickdip-stained blood smears, and no further parasite-positive wells were identified.

### *In vitro* P. falciparum growth inhibition assays.

*In vitro* inhibition of P. falciparum growth was assessed using a 72-h isotopic microtest, essentially as previously described (43). Briefly, highly synchronous ring-stage P. falciparum-infected erythrocytes obtained by sorbitol treatment (42) were seeded at 0.5% parasitemia and 2.5% final hematocrit into 96-well tissue culture plates (3596 Costar; Corning, USA) containing serial dilutions of control or test compounds. Compound vehicle only (0.5% final DMSO) and the antimalarial drug chloroquine served as negative and positive controls, respectively, in each assay. After incubating for 48 h under standard P. falciparum culture conditions in RPMI 1640 medium (Gibco, USA) containing 10% heat-inactivated pooled human sera and 5 μg/ml gentamicin (Sigma, USA), 0.5 μCi [^3^H]-hypoxanthine (PerkinElmer, USA) was added to each well followed by culturing for a further 24 h. Cells were harvested onto 1450 MicroBeta filter mats (Wallac, USA), and ^3^H incorporation was determined using a 1450 MicroBeta liquid scintillation counter (PerkinElmer). Percentage inhibition of growth for compound treated versus that for matched vehicle only (0.5% DMSO) controls was determined, and IC_50_ values were calculated using nonlinear regression analysis in GraphPad Prism. Each compound was assayed in triplicate wells in at least three independent experiments. Statistical difference between IC_50_s was determined using an unpaired *t* test with GraphPad Prism data analysis software.

### *In vitro* P. falciparum resistance selection.

P. falciparum 3D7 clone C3 (3D7-C3)-infected erythrocytes were cultured with (and, in parallel, without) PS-3 at 1× IC_50_ (0.9 μM). Parasite growth was monitored every 1 to 3 days via microscopic examination of Quickdip-stained (POCD, Australia) thin blood films, with medium changed as needed. When PS-3-treated parasites were observed to be replicating at a similar rate to that of the untreated controls, compound pressure was gradually increased in a stepwise manner over several weeks until the parasites were surviving in 10× IC_50_ concentrations. At this point, the selected and wild-type clones were assessed in growth inhibition assays to determine sensitivity for PS-3 and other compounds. Once a resistance phenotype was confirmed, PS-3-selected and wild-type parasites were subcloned and phenotype assessed again to confirm resistance.

### *In vitro* growth rate analysis of P. falciparum.

Highly synchronous ring-stage P. falciparum cultures starting at 0.25% rings and 2.5% hematocrit were cultured under standard conditions for 72 h. Quickdip-stained (POCD, Australia) thin blood films were prepared every 24 h, and ∼3,000 erythrocytes were counted by two independent microscopists to determine the mean number of parasites infecting 100 erythrocytes at each time point. Three independent assays were assessed per clone.

### Genome sequencing.

Genomic DNA was isolated from P. falciparum-infected erythrocytes using a DNeasy blood and tissue kit (Qiagen, USA). The Nextera XT kit (Illumina) was used to prepare DNA libraries from samples for whole-genome sequencing using the dual index protocol. The libraries were run on the Illumina HiSeq 2500 in rapid run mode with 100-bp paired-end reads. The reads were aligned to the P. falciparum 3D7 reference genome (PlasmoDB v. 13.0) as described previously (44). Single nucleotide polymorphisms (SNPs) and indels were called with the Genome Analysis Toolkit’s (GATK) HaplotypeCaller (45, 46). Variants were filtered by quality scores and sequencing bias statistics based on GATK’s default filtering parameters. SNPs were filtered out if they met any of the following criteria: quality depth (QD), <2.0; mapping quality (MQ), <50.0, Phred-scaled *P* value using Fisher’s exact test to detect strand bias (FS), >60.0; symmetric odds ratio (SOR), >4.0; Z-score from Wilcoxon rank sum test of alternative versus reference read mapping qualities (MQRankSum), less than −12.5; ReadPosRankSum (RPRS) parameter, less than −8.0. Indels were filtered out if they met any of the following criteria: QD, <2.0; RPRS, less than −20.0; FS, >200.0. Variants were annotated using snpeff (version 4.2) (47). Custom scripts were used to compare the variants between the parent sequence and the resistant clones.

### CRISPR-Cas9 genome editing.

CRISPR/Cas9 editing of mutations in *Pf*PFK9 was performed using a pDC2-based Cas9 guide RNA (gRNA) plasmid, pDC2-cam-coCas9-U6.2-hDHFR. Two sgRNAs were designed per target site using Benchling (San Francisco, CA). For targeting of L513, gRNA1 (CAATTTATGTCACATTATCT) and gRNA2 (TCACATTATCTAGGTTATGA) were employed, and for targeting of R860, gRNA3 (CATAACACATTCATAGCACC) and gRNA4 (GGTGCTATGAATGTGTTATG) were used. Donor templates with 656-bp or 750-bp homology to the L513 and R860 target sites, respectively, were synthesized (Thermo Fisher) and cloned into the AatII-EcoRI sites of the Cas9 vector using Gibson assembly. Plasmids were transfected by electroporation (0.31 kV, 950 μF) into Dd2 parasites and selected with 2.5 nM WR99210 for 8 days before drug pressure was removed. Editing of the recovered parasites was examined by Sanger sequencing of the bulk culture, and clonal lines were derived by limiting dilution.

### Metabolite profiling and stable isotope labeling.

Erythrocytes infected with 3D7-C3^PS3^ clones 1 and 3 and 3D7-C3 were regularly sorbitol synchronized, and trophozoites were separated from uninfected erythrocytes using a magnet supplied by Colebrook Bioscience. The enriched infected erythrocytes (>95% parasitemia) were then allowed to recover for 0.5 to 1 h at 37°C in “complete medium” (RPMI 1640 supplemented with 0.5% AlbuMAX II, 5% human serum, 20 mM glucose [final concentration], 25 mM HEPES, 100 μM hypoxanthine, and 10 μg/ml gentamicin).

Stable-isotope incorporation was performed using methods previously described with minor modifications (48). Briefly, purified infected erythrocytes were resuspended in fresh RPMI 1640 medium at a cellular density of 1 × 10^8^ cells/ml and allowed to recover for 10 min. Time courses were initiated by adding an equal volume of RPMI 1640 containing 11 mM ^13^C-U-glucose (Sigma), leading to a 1:1 mix of fully unlabeled/fully labeled glucose. This 1:1 mixing was performed to avoid perturbation of metabolism during label addition and allows detection of more complex labeling patterns. At predetermined time points, 1 × 10^8^ cells were aliquoted, centrifuged (15 s at 14,000 × *g*), washed with 1 ml ice-cold phosphate-buffered saline (PBS), and centrifuged (15 s at 14,000 × *g*), and metabolites were extracted with 200 μl of 80% acetonitrile (containing 1 μM ^13^C-U-aspartate). Samples were rapidly vortexed and centrifuged (5 min 14,000 × *g*), and the supernatant was collected.

The metabolites were separated on a SeQuant ZIC-pHILIC column (5 μM, 150 mm by 4.6 mm; Millipore) with a 1260 series high-pressure liquid chromatography (HPLC) system (Agilent) using a method previously described with modifications (49). Briefly, a flow rate of 0.3 ml/min was used with 20 mM ammonium carbonate in water (A) and 100% acetonitrile (B) as the mobile phase. A binary gradient was set up as follows: 0.5 min, 80% B; 15.5 min, 50% B; 17.5 min, 20% B; 18.5 min, 5% B; 21 min, 5% B; 23 min, 80% B; held at 80% B until 29.5 min. Detection of metabolites was performed on an Agilent Q-TOF mass spectrometer 6545 operating in negative electrospray ionization (ESI) mode. The scan range was 85 to 1,200 *m/z* between 2 and 27 min at 0.8 spectra/second.

LC-MS .d files were converted to .mzXML files using MS convert and analyzed using MAVEN (50). Following alignment, metabolites were assigned using exact mass (<10 ppm) and retention time (compared to a standards library of 150 compounds run the same day). Isotopologues for each metabolite of interest were extracted and integrated, and percent ^13^C incorporation was converted into concentration of metabolite labeled using the absolute metabolite concentrations determined previously (48). When metabolite values were not previously determined (ribose-5-P, sedoheptulose-7-P, octulose-8-P, and malate), metabolite concentrations were arbitrarily set to 100 μM.

The drug-induced changes to the parasite metabolite profile were determined using the experimental approach described in reference 49. Drug was added at 40 μM to cell suspensions (each containing 1 × 10^8^ cells at 0.4% hematocrit), which were incubated at 37°C under controlled atmospheric conditions (5% CO_2_ and 1% O_2_ in N_2_). After 2 h of incubation, cell suspensions were processed and metabolites were extracted for LC-MS analysis as described above.

### Isopentenyl pyrophosphate rescue.

*In vitro* isopentenyl pyrophosphate (IPP) pathway rescue against P. falciparum 3D7 parasites was carried out as previously described (39). Briefly, PS-3 was tested in P. falciparum in a 72-h [^3^H]hypoxanthine growth inhibition assay as previously described (43), with the following modifications. Two identical 72-h assays were performed simultaneously, one supplemented with 200 μM IPP and one without IPP. The antibiotic antimalarial compound clindamycin was used as a positive control. Three or more independent assays were performed, each in triplicate wells. Each compound was assayed in triplicate wells in at least three independent experiments. Statistical difference between IC_50_s was determined using a two-tailed *t* test with GraphPad Prism data analysis software.

### Phosphatase activity of *Pf*HAD1 and *Pf*HAD2.

Recombinant enzymes *Pf*HAD1 and *Pf*HAD2 were expressed and purified fresh as previously described in references 9 and 7, respectively. Phosphate release was quantified using the EnzChek phosphate assay kit (Invitrogen, Life Technologies) as previously described (8). In all assays, 200 ng of *Pf*HAD1 and 2,000 ng of *Pf*HAD2 were used and determined to be within the linear range for assay sensitivity (data not shown). Kinetic parameters for phosphate-containing substrate AMP (Sigma) were determined from three independent *K_m_* curves for each enzyme, with nonlinear regression analysis performed using GraphPad Prism. The *K_m_*s for AMP were determined to be 4.11 ± 0.88 mM and 4.09 ± 0.55 mM for *Pf*HAD1 and *Pf*HAD2, respectively. Inhibition of phosphatase activity for compounds PS-3, PS-3′, and PS-12 was tested across a range of inhibitor concentrations (200 μM to 3 nM) at an ATP concentration of 4 mM. Nonlinear regression was attempted for inhibition curves using GraphPad Prism; unresolved “ambiguous” fitted curves indicate lack of inhibition up to 200 μM under the described assay conditions.

### PFK recombinant protein expression.

Recombinant proteins *Pk*PFKα (762 to 1417 amino acids [aa]), *Pk*PFKβ (1 to 663 aa), *Pf*PFKα (778 to 1418 aa), and *Pf*PFKβ (1 to 663 aa) were codon optimized by Genewiz and cloned between the NdeI and BamHI cloning sites of plasmid BG1861, which introduces an N-terminal 6×His tag. A catalytically dead mutant, *Pf*PFKβΔKTIDGD, was also generated utilizing Q5 site-directed mutagenesis (NEB Inc.). Constructs were transformed into the BL21(DE3) Escherichia coli expression strain (Life Technologies). Cultures were grown to an optical density at 600 nm (OD_600_) of ∼0.6 in the presence of ampicillin (100 μg/ml) at 37°C shaking at 200 rpm and induced for 2 h with isopropyl-β-d-thiogalactoside. Cells were collected by centrifugation and stored at −80°C. Pellets were resuspended in sonication lysis buffer containing 10 mM Tris-HCl (pH 7.5), 20 mM imidazole, 1 mM MgCl_2_, 1 mM dithiothreitol (DTT), 1 mg/ml lysozyme, 100 U Benzonase and cOmplete Mini EDTA-free protease inhibitor tablets (Roche Applied Science). Proteins were purified via nickel agarose beads (Gold Biotechnology) and eluted with 300 mM imidazole, 20 mM Tris-HCl (pH 7.5), and 150 mM NaCl. Eluted proteins were further purified via size exclusion chromatography using a HiLoad 16/60 Superdex 200 gel filtration column (GE Healthcare Life Sciences) using an AKTA Explorer 100 fast protein liquid chromatograph (FPLC) (GE Healthcare Life Sciences). Fast protein liquid chromatography buffer contained 100 mM Tris-HCl (pH 7.5), 1 mM MgCl_2_, 1 mM DTT, and 10% (wt/vol) glycerol. Fractions containing purified protein were pooled, concentrated to ∼2 mg/ml as determined via Pierce bicinchoninic acid (BCA) protein assay kit (Thermo Fisher), and flash frozen using liquid nitrogen (LN_2_) for storage at −80°C.

### PFK recombinant assays.

Recombinant PFK activity was measured using a linked enzyme assay as previously described (5, 7, 51). Briefly, reactions contained 100 mM Tris-HCl (pH 7.5), 1 mM MgCl_2_, 1 mM DTT, 10% (wt/vol) glycerol, 0.25 mM NADH, 1 mM ATP, 3 mM fructose 6-phosphate, and excess of linking enzymes aldolase (7.5 U), triose-phosphate isomerase (3.8 U), and glycerol 3-phosphate dehydrogenase (3.8 U). Activity in the presence of inhibitors (50 μM) was tested for each recombinant protein and normalized to that for no inhibitor solvent-containing positive controls. Inhibition curves for compounds PS-3 and PS-12 were determined for *Pf*PFKβ across the 1:2 dilution series comprising the concentration range 100 μM to 97 nM. Inhibition curves were fitted using nonlinear regression analysis using GraphPad Prism; unresolved curves indicate minimum IC_50_ values.

### *Pf*PFK model construction.

*Pf*PFK subunits were searched against the HHpred server for protein remote homology detection and three-dimensional (3D) structure prediction using statistics as previously described (52–55). The Borreliella burgdorferi PFK structure (PDB 1KZH [56]) returned the highest similarity for both *Pf*PFK domains and was used to predict the 3D structure for each domain using the program MODELLER. PFK product orientation in the active site of the model was predicted via the alignment tool, using PyMOL software against the E. coli PFK crystal structure (PDB 1PFK [57]).
