# Supplementary material for: The Key Glycolytic Enzyme Phosphofructokinase Is Involved in Resistance to Antiplasmodial Glycosides
Source: mBio. 2020 Dec 8;11(6):e02842-20. doi: 10.1128/mBio.02842-20 (PMC7733947; doi:10.1128/mBio.02842-20)
Supplement: FIG S1 [file mBio.02842-20-sf001.pdf]

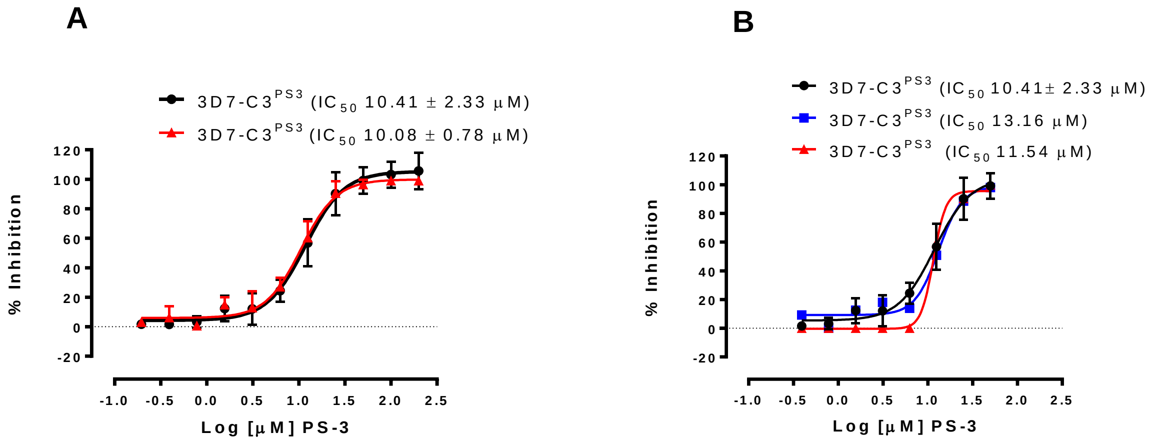

Fig S1: *P. falciparum* 3D7-C3^{PS3} displays a stable phenotype. (**A**) The sensitivity of *P. falciparum* 3D7-C3^{PS3} to **PS-3** prior to (black line) and after (red line) cryopreservation was assessed using 72h ^{3}H-Hypoxanthine uptake growth inhibition assays. Mean percentage inhibition (±SD) is shown for three independent assays, each carried out in triplicate wells. (**B**) The sensitivity of *P. falciparum* 3D7-C3^{PS3} to **PS-3** following withdrawal from **PS-3** pressure for 4 weeks (blue line) and 10 weeks (red line) was assessed using 72h ^{3}H-Hypoxanthine uptake growth inhibition assays and compared to the sensitivity of *P. falciparum* 3D7-C3^{PS3} exposed continually to **PS-3** (10µM; black line).
