# Supplementary material for: The Key Glycolytic Enzyme Phosphofructokinase Is Involved in Resistance to Antiplasmodial Glycosides
Source: mBio. 2020 Dec 8;11(6):e02842-20. doi: 10.1128/mBio.02842-20 (PMC7733947; doi:10.1128/mBio.02842-20)
Supplement: FIG S2 [file mBio.02842-20-sf002.pdf]

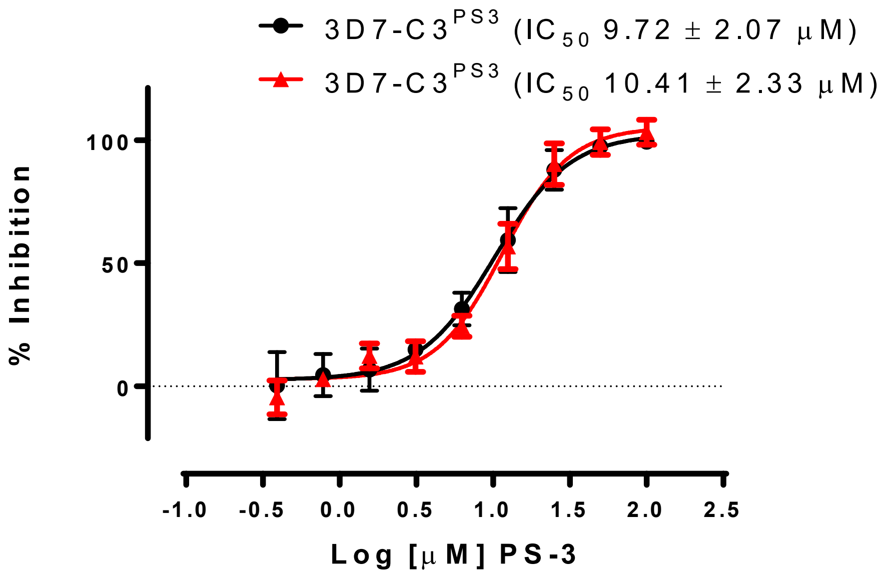

Fig S2: Exposure of 3D7-C3^{PS3} to ~20x PS-3 IC$_{50}$ does not significantly alter PS-3 activity. The sensitivity of *P. falciparum* 3D7-C3^{PS3} to **PS-3** at ~10x IC$_{50}$ (10μM; red line) and ~20x IC$_{50}$ (20μM; black line) was assessed using 72h ^{3}H-Hypoxanthine uptake growth inhibition assays. Mean percentage inhibition (±SD) is shown for three independent assays, each carried out in triplicate wells. Increasing **PS-3** exposure from 10 μM to 20μM did not result in any significant difference in 3D7-C3^{PS3} **PS-3** IC$_{50}$ (P >0.05).
