# Supplementary material for: The Key Glycolytic Enzyme Phosphofructokinase Is Involved in Resistance to Antiplasmodial Glycosides
Source: mBio. 2020 Dec 8;11(6):e02842-20. doi: 10.1128/mBio.02842-20 (PMC7733947; doi:10.1128/mBio.02842-20)
Supplement: FIG S3 [file mBio.02842-20-sf003.pdf]

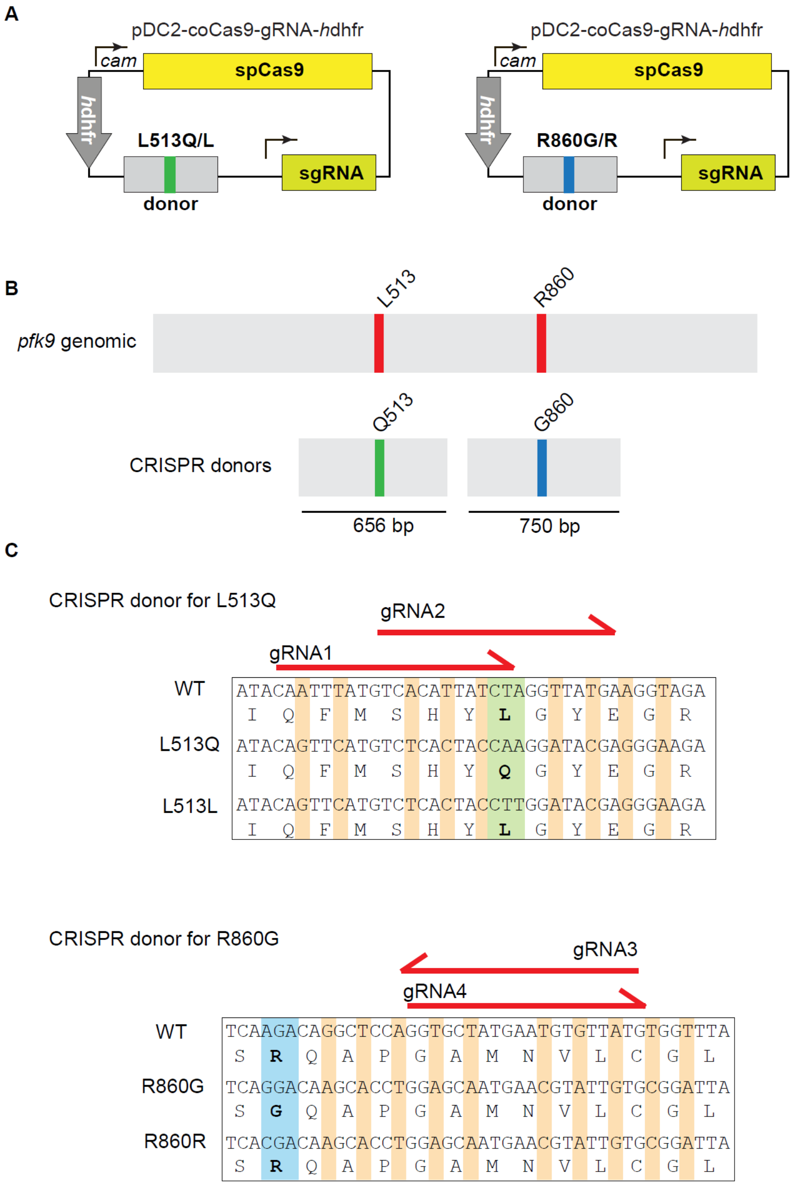

Fig S3: CRISPR/Cas9 genome editing of PfPFK9.

A) Schematic of the Cas9-gRNA-donor plasmids for either the L513Q and L513-silent donor (left) or the R860G and R860-silent donor (right). **B**) Genomic PfPFK9 target site and the donor homology regions of 656 bp (L513Q) and 750 bp (R860G) of the synthesised donor templates. **C**) Sequence of a region of the CRISPR donors, showing the gRNA binding sites and the desired mutations. Additional silent binding-site mutations (orange) were included in all donors to prevent gRNA binding and Cas9 cleavage of the edited genome.
