# Supplementary material for: The Key Glycolytic Enzyme Phosphofructokinase Is Involved in Resistance to Antiplasmodial Glycosides
Source: mBio. 2020 Dec 8;11(6):e02842-20. doi: 10.1128/mBio.02842-20 (PMC7733947; doi:10.1128/mBio.02842-20)
Supplement: FIG S4 [file mBio.02842-20-sf004.pdf]

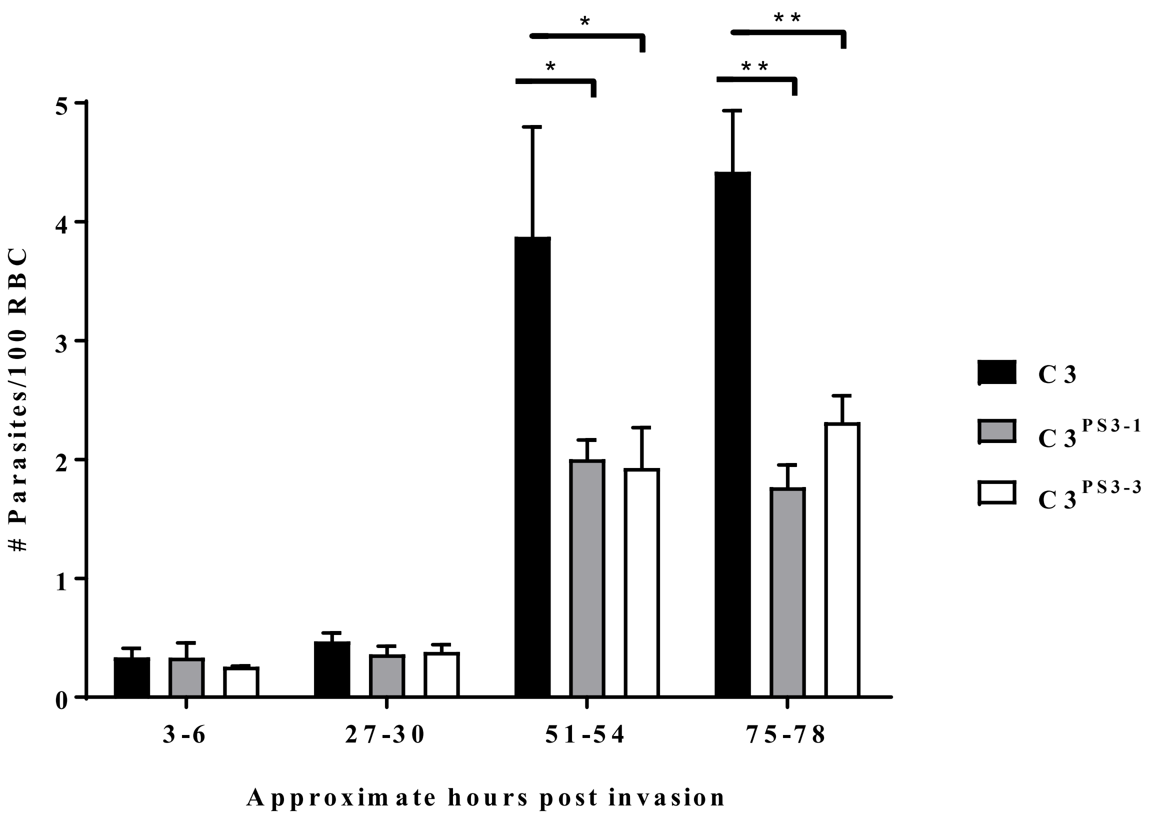

Fig S4: *In vitro* growth analysis of *P. falciparum* 3D7-C3^{PS3} clones versus 3D7-C3 wild type parasites. Growth of 3D7-C3^{PS3-1}, 3D7-C3^{PS3-3} and 3D7-C3 *P. falciparum* parasites over 72h (starting at ~3-6h post invasion) was determined by microscopic examination of Quickdip stained thin blood smears taken every 24h. Mean number of parasites per 100 RBCs cells was determined at each time point by examining >3,000 infected red blood cells (RBCs) by two independent microscopists. Results are mean (±SD) from three independent experiments. *P<0.05; **P<0.01.
