# Supplementary material for: The Key Glycolytic Enzyme Phosphofructokinase Is Involved in Resistance to Antiplasmodial Glycosides
Source: mBio. 2020 Dec 8;11(6):e02842-20. doi: 10.1128/mBio.02842-20 (PMC7733947; doi:10.1128/mBio.02842-20)
Supplement: FIG S5 [file mBio.02842-20-sf005.pdf]

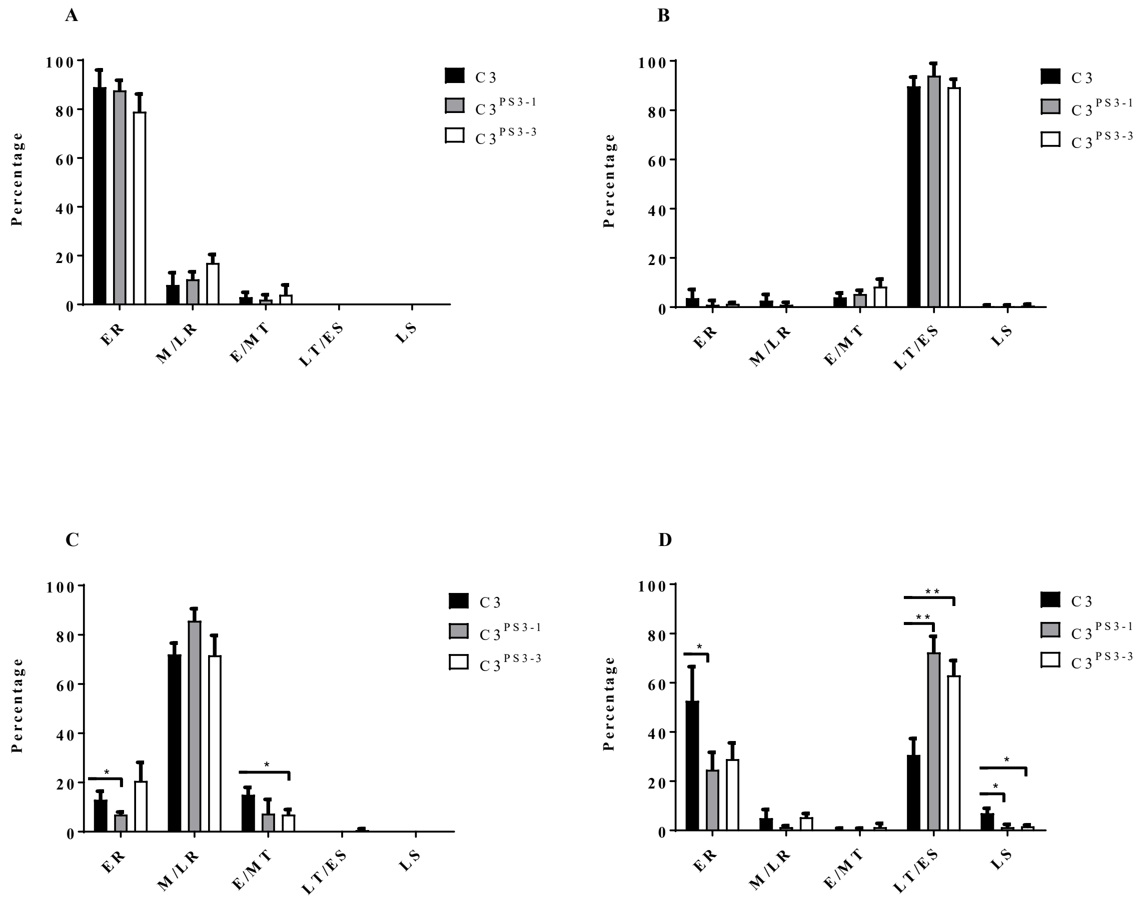

Fig S5: *In vitro* developmental stage analysis of asexual intraerythrocytic *P. falciparum* 3D7-C3^{PS3} clones versus 3D7-C3 wild type parasites. Different asexual intraerythrocytic developmental stages of 3D7-C3^{PS3-1}, 3D7-C3^{PS3-3} and 3D7-C3 *P. falciparum* parasites were assessed at 3-6h (**A**), 27-30h (**B**), 51-54h (**C**) and 75-78h (**D**) post invasion (>150 parasites counted per time point). Data are the mean (±SD) of each developmental form as a percentage of the total number of parasites for three independent assays. ER, early ring; M/LR, mid to late ring; E/MT, early to mid trophozoite; LT/ES, late trophozoite to early schizont; LS, late schizont. *P<0.05; **P<0.01.
