# Supplementary material for: The Key Glycolytic Enzyme Phosphofructokinase Is Involved in Resistance to Antiplasmodial Glycosides
Source: mBio. 2020 Dec 8;11(6):e02842-20. doi: 10.1128/mBio.02842-20 (PMC7733947; doi:10.1128/mBio.02842-20)
Supplement: FIG S6 [file mBio.02842-20-sf006.pdf]

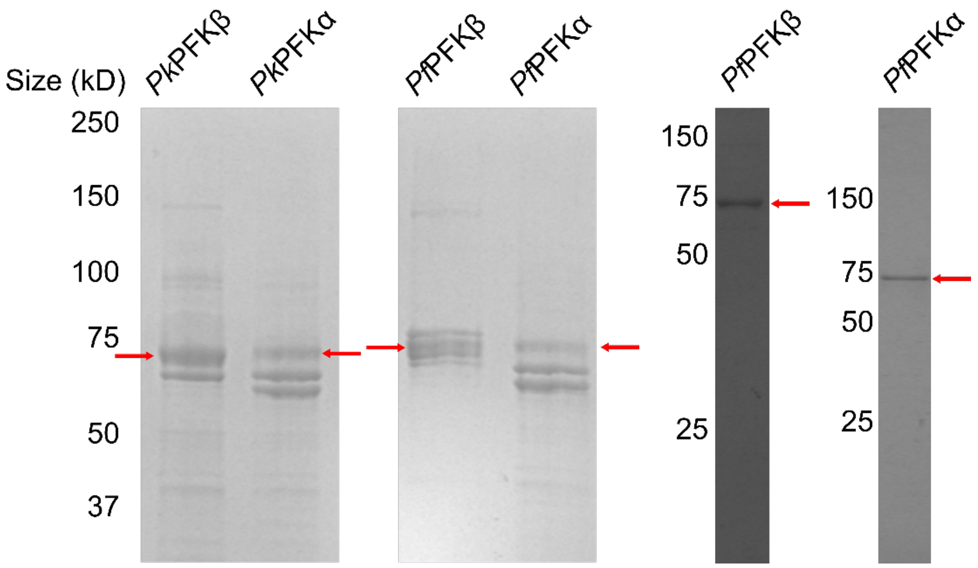

Fig S6. Coomassie gel images of purified PFK orthologs. Left, heterologous protein expression of *Plasmodium knowlesi* and *Plasmodium falciparum* alpha and beta subunits (designated by red arrows). Protein identity was confirmed by expected band size and mass spectrometry. Right, *Pf*PFK subunits following additional purification using size exclusion chromatography.
