# Supplementary material for: The Key Glycolytic Enzyme Phosphofructokinase Is Involved in Resistance to Antiplasmodial Glycosides
Source: mBio. 2020 Dec 8;11(6):e02842-20. doi: 10.1128/mBio.02842-20 (PMC7733947; doi:10.1128/mBio.02842-20)
Supplement: FIG S7 [file mBio.02842-20-sf007.pdf]

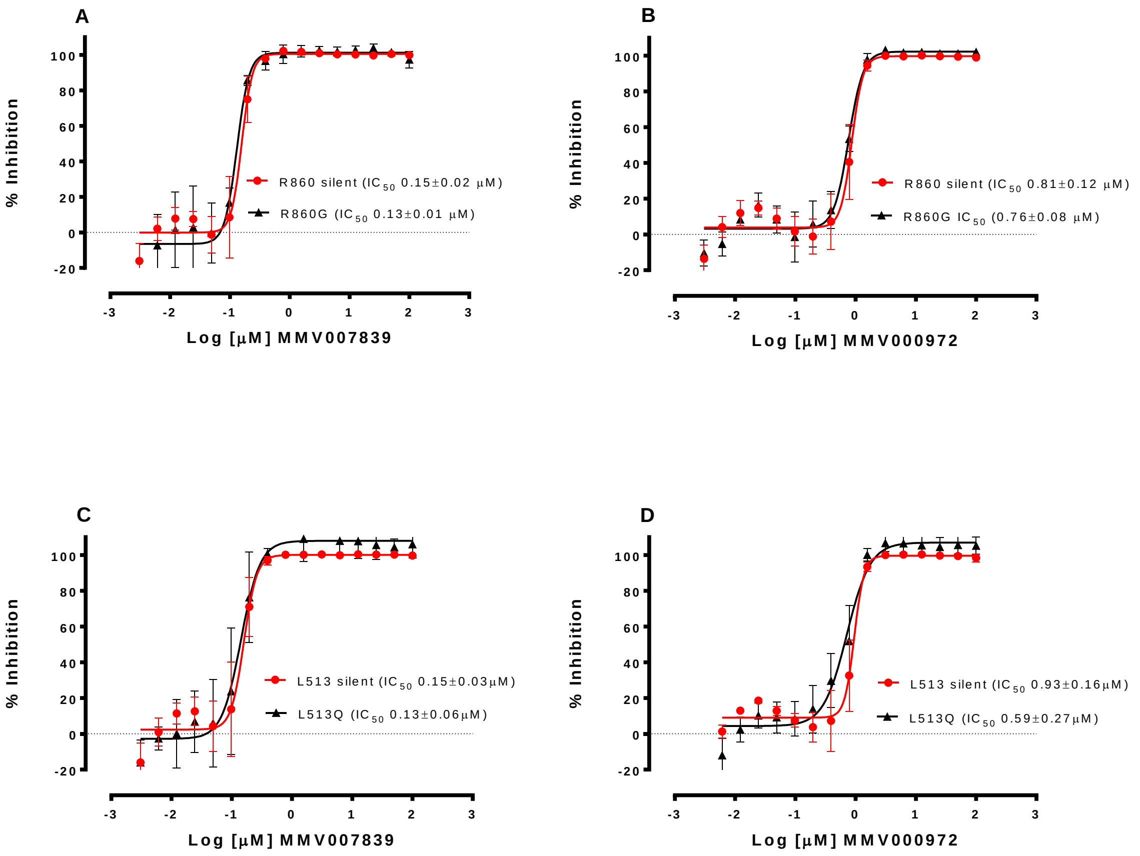

Fig S7: *In vitro* profile of *P. falciparum* PfPFK9 mutant parasites against PfFNT inhibitors. The mean percent growth inhibition (±SD) of *P. falciparum* PfPFK9 mutant lines and controls R860G/R860 silent (**A** and **B**) and L513Q/ L513 silent (**C** and **D**) against the PfFNT inhibitors MMV007839 (**A** and **C)** and MMV000972 (**B** and **D)** was assessed using 72h ^{3}H-Hypoxanthine uptake growth inhibition assays. In each case, four independent assays, each in triplicate wells, were carried out and mean (±SD) 50% Inhibitory concentrations (IC_{50s}) determined using non - linear regression analysis in GraphPad prism®.
