# Supplementary material for: The Key Glycolytic Enzyme Phosphofructokinase Is Involved in Resistance to Antiplasmodial Glycosides
Source: mBio. 2020 Dec 8;11(6):e02842-20. doi: 10.1128/mBio.02842-20 (PMC7733947; doi:10.1128/mBio.02842-20)
Supplement: FIG S8 [file mBio.02842-20-sf008.pdf]

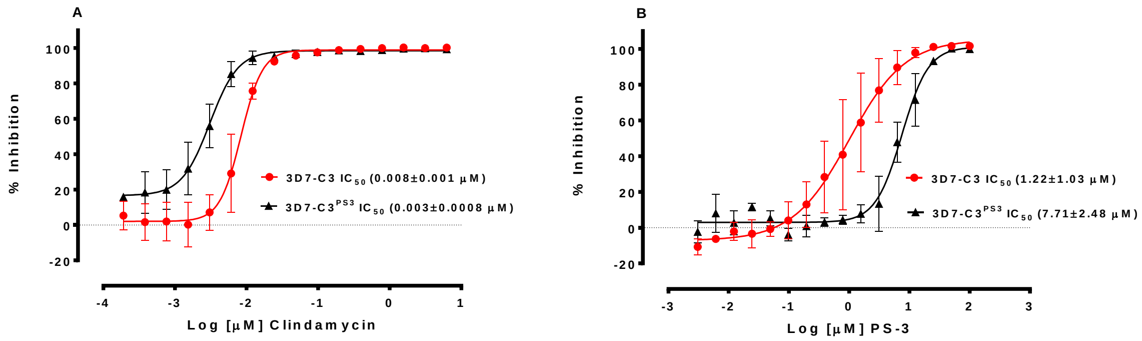

Fig S8: *In vitro* profile of *P. falciparum* PS-3 resistant parasites against clindamycin. The mean percent growth inhibition (±SD) of *P. falciparum* 3D7-C3^{PS3} and 3D7-C3 parasites against clindamycin (**A**) and the control compound **PS-3** (**B**) was assessed using 96h ^{3}H-Hypoxanthine uptake growth inhibition assays. In each case 2 independent assays, each in triplicate wells, were carried out and mean (±SD) 50% Inhibitory concentrations (IC_{50s}) determined using non - linear regression analysis in GraphPad prism®.
