# Supplementary material for: The Key Glycolytic Enzyme Phosphofructokinase Is Involved in Resistance to Antiplasmodial Glycosides
Source: mBio. 2020 Dec 8;11(6):e02842-20. doi: 10.1128/mBio.02842-20 (PMC7733947; doi:10.1128/mBio.02842-20)
Supplement: FIG S9 [file mBio.02842-20-sf009.pdf]

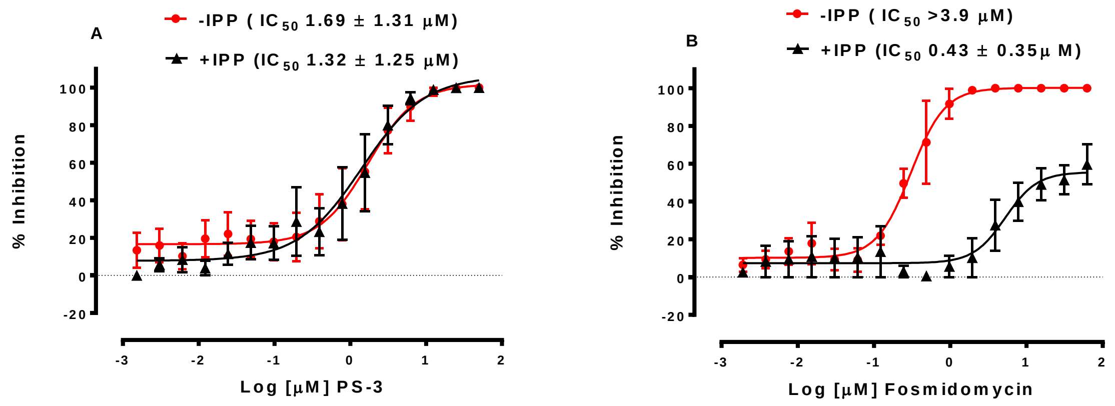

Fig S9: PS-3 *in vitro* activity is not rescued by IPP supplementation. The sensitivity of *P. falciparum* 3D7 against compound **PS-3** (**A**) and fosmidomycin (**B**) without IPP (red lines) and with 200µM IPP (black lines) was assessed using 72h ^{3}H-Hypoxanthine uptake growth inhibition assays. In each case the mean percent inhibition (±SD) compared to DMSO controls was determined for three independent assays, each carried out in triplicate wells. Mean (±SD) 50% Inhibitory concentrations (IC_{50s}) determined using non - linear regression analysis in GraphPad prism®.
